# Optimized Electrodeposition of Ni_2_O_3_ on Carbon Paper for Enhanced Electrocatalytic Oxidation of Ethanol

**DOI:** 10.1021/acsomega.4c01658

**Published:** 2024-06-29

**Authors:** Ruixing Du, Qitong Zhong, Xing Tan, Longfei Liao, Zhenchen Tang, Shiming Chen, Dafeng Yan, Xuebin Zhao, Feng Zeng

**Affiliations:** † State Key Laboratory of Materials-Oriented Chemical Engineering, College of Chemical Engineering, Nanjing Tech University, Nanjing 211816, Jiangsu, China; ‡ School of Materials Science and Engineering, Harbin Institute of Technology (Shenzhen), Shenzhen 518055, Guangdong, China; § School of Intelligent Medicine, China Medical University, Shenyang 110122, Liaoning, China; ∥ College of Chemistry and Chemical Engineering, Hubei University, Wuhan 430062, China; △ Technology Center, China Tobacco Henan Industrial Co., Ltd., Zhengzhou 450000, China

## Abstract

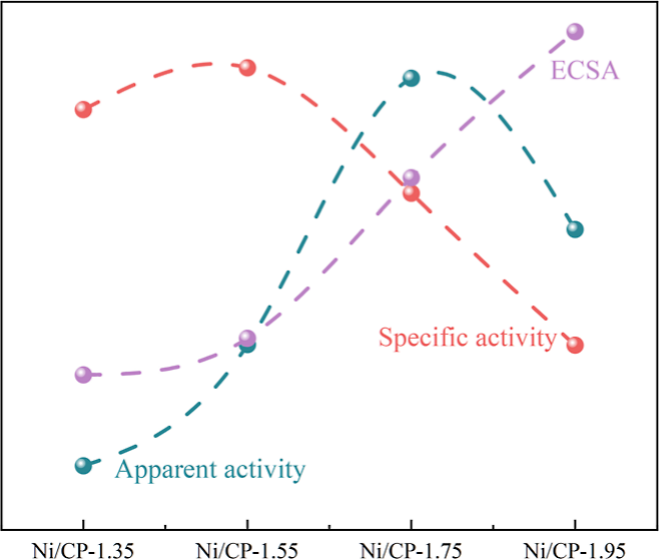

The urgent need for sustainable and efficient energy conversion technologies has propelled research into novel electrocatalysts for fuel cell applications. This study investigates a carbon paper (CP)-supported Ni_2_O_3_ catalyst for the electrocatalytic oxidation of ethanol. We utilized electrodeposition to uniformly deposit/dop Ni_2_O_3_ onto the CP, creating an effective electrocatalyst. Our approach allows the tailoring of the doping degree by adjusting the electrodeposition potential. The optimal doping degree, achieved at a medium deposition potential, results in an electrode with high intrinsic activity and a substantial electrochemically active surface area (ECSA), thereby enhancing its electrocatalytic activity. This catalyst efficiently facilitates the oxidation of ethanol to formic acid while maintaining good stability. The enhanced performance is attributed to the effective interface and interaction between Ni_2_O_3_ and CP. This work not only provides insights into the design of efficient Ni-based catalysts for ethanol oxidation but also paves the way for developing advanced materials for renewable energy conversion.

## 1 Introduction

In response to the escalating challenges posed by fossil energy consumption and environmental pollution, there has been a heightened focus on the development and adoption of novel and environmentally friendly energy sources.^1−3^ Ethanol, characterized as a biomass derived liquid fuel, stands out as a promising renewable energy option and a key green chemical raw material. It has also emerged as a viable alternative to H_2_.^4,5^ The advantages of ethanol as a fuel are evident; it boasts enhanced safety during transportation and storage compared to H_2_, exhibits low toxicity relative to other alcohols, and its cost-effective production through the fermentation of sugar-containing raw materials is well-established.^6,7^ Furthermore, ethanol possesses a high energy density, with complete oxidation yielding 8.0 kW h kg^–1^,^8,9^ rendering ethanol fuel cells a compelling prospect for renewable electrochemical energy conversion.^10^

However, the efficiency of ethanol fuel cells relies heavily on the electrocatalytic processes occurring at the anode, necessitating effective electrocatalysts to minimize electrochemical overpotential and achieve optimal ethanol energy conversion.^11,12^ Presently, Pt materials dominate as the most efficient electrocatalysts for ethanol oxidation reactions.^13^ Despite their efficiency, the drawbacks of Pt, including high cost, limited reserves, and susceptibility to poisoning by CO or CHO species, hinder their widespread industrial application.^4,14^ Consequently, the pursuit of non-Pt electrocatalysts with high catalytic activity has become a prominent avenue of research in recent years.

Transition metals have emerged as promising candidates for anode catalysts in ethanol oxidation reactions due to their low cost, high poisoning tolerance, and notable activity.^13,15^ Among transition metals, Ni-based materials, such as NiO and Ni(OH)_2_ have proved their suitability in various energy-related applications, ranging from supercapacitors and hydrogen evolution reaction to alcohol oxidation reaction, owing to nickel’s numerous benefits—including high catalytic surface activity, excellent electrical conductivity, widespread availability, affordability, and chemical stability.^7,16^ Moreover, the catalytic influence of Ni on ethanol oxidation is notably more pronounced than that of other metals like Co and Fe, as well as their respective oxides.^17^ Ni-based materials have shown increasing potential in realizing highly efficient electrochemical ethanol oxidation.^7,18^ Besides, various strategies such as tailoring the nature of Ni species, supporting Ni species on carbon materials, applying advanced synthesis methods, and using promoters have been developed to increase the electrocatalytic performance.^18^ Various Ni species have been studied for ethanol oxidation. For instance, Ni-nanoparticle-doped carbon composite shows a peak current density of 47 mA cm^–2^,^19^ and Ni(OH)_2_ aerogel catalysts show a peak current density of 27.6 mA cm^–2^.^20^ Moreover, Shekhawat et al. embedded NiO_*x*_ in nitrogen doped carbon nanosheets, achieving 10 mA cm^–2^ at 1.354 V vs RHE.^21^ Although the electrochemical performance of different Ni species varies, the ethanol oxidation reaction depends on the reversible conversion of Ni (II)/Ni (III). It has been proved that Ni (hydr) oxides as a precursor, transform to Ni(II)/Ni(III) on immersing into the electrolyte and applying a potential, and the Ni(II)/Ni(III) redox couple serves as the primary catalytic mechanism for ethanol electro-oxidation in Ni-based materials.^18^ Therefore, the precursor, which may influence the formation of Ni(II)/Ni(III), plays a key role in electrocatalytic oxidation of ethanol. It has been previously proved that Ni_2_O_3_ is a promising catalyst for electrochemical oxidation of urea with Ni^3+^ ions being highly active. Moreover, Ni_2_O_3_ possess better tolerance toward CO_*x*_ poisoning, leading to high stability.^22^ Safeer et al. discovered that Ni_2_O_3_ is more reactive to hydroxylation than NiO, which is attributed to the active Ni (III) sites on the Ni_2_O_3_ surface. The adsorption energy for OH on the pristine NiO surface was found to be −0.75 eV per OH group, in contrast to −0.98 eV for the pristine Ni_2_O_3_ surface. This indicates stronger adsorption of OH on the Ni_2_O_3_ surface. Furthermore, the research highlights the enhanced efficacy of Ni (III) in the oxidation of urea.^22^ However, using Ni_2_O_3_ as a precursor for catalysts of electrochemical ethanol oxidation has not yet been investigated.

Furthermore, early studies have investigated the combination of carbon materials as a support and Ni as an electrocatalyst for ethanol oxidation. Plascencia et al. pioneered the preparation of a highly active carbon-supported Ni electrocatalyst through a hydrothermal method.^23^ Carbon paper (CP), with its specific properties, electrochemical activity, porous structure, and electrical conductivity, has become a favored material for this reaction.^24^

Against this backdrop, our work delves into the exploration of a CP-supported Ni_2_O_3_ catalyst for the electrocatalytic oxidation of ethanol. Utilizing CP as a support, we employed electrodeposition to uniformly deposit/dop Ni_2_O_3_ onto the CP surface, creating an effective electrocatalyst for ethanol oxidation. By tailoring the electrodeposition potential, the doping degree can be readily tailored. The optimized doping degree obtained with medium deposition potential leads to both high specific current density, and electrochemically active surface aera (ECSA) leads to high electrocatalytic activity for ethanol oxidation to formic acid together with good stability. The interface/interaction between Ni_2_O_3_ and CP are responsible for the enhancement.

## 2 Experimental Section

### 2.1 Electrode Preparation

Ni_2_O_3_ was electrochemically deposited on CP using a CS310X electrochemical workstation. Initially, Ni(NO_3_)_2_·6H_2_O (7.27 g) was dissolved in 50 mL of ultrapure water, constituting the electrolyte for electrodeposition. A three-electrode system, working electrode: CP (Hesen, HCP120, 1 × 0.44 cm), counter electrode: graphite rod, reference electrode: Ag/AgCl electrode filled with saturated KCl, was employed for constant potential polarization for 3600 s at potentials of 1.35, 1.55, 1.75, and 1.95 V versus Ag/AgCl. The deposition current density over time is presented in Figure S1. Subsequently, the working electrode underwent rinsing with ultrapure water. The resulting electrodes were denoted as Ni/CP-1.35, Ni/CP-1.55, Ni/CP-1.75, and Ni/CP-1.95, corresponding to different deposition potentials.

### 2.2 Characterization of Electrodes

Ni content on CP was measured by inductively coupled plasma optical emission spectrometry (ICP-OES, Agilent 5110). X-ray diffraction (XRD) was analyzed by using a SmartLab diffractometer equipped with a Cu Kα source (λ = 1.5406 Å, 40 kV, and 15 mA). XPS analysis was carried out on an Escalab 250Xi spectrometer equipped with a Mg Kα X-ray source. The binding energies of the spectra were calibrated by using the C 1s peak at 284.8 eV as a reference. Scanning electron microscopy (SEM) images were captured with a Regulus 8100 electron microscope at a potential of 5.00 kV, and energy dispersive X-ray spectroscopy (EDS) element-mapping images were acquired using a smartedx detector. Raman spectra were recorded by employing a HORIBA HR Evolution spectrometer with a 532 nm YAG solid-state laser.

### 2.3 Electrochemical Measurements

The electrochemical oxidation of ethanol was performed using a CS310X electrochemical workstation connected to a three-electrode system which consisted of a counter electrode, a reference electrode, and a working electrode. The material of the counter electrode is a graphite rod; the reference electrode is an Ag/AgCl electrode (filled with saturated KCl solution); and the working electrode is the prepared Ni/CP electrode. These experiments were carried out at room temperature in 1 M KOH and 1 M EtOH solutions. The potential scan range of cyclic voltammetry (CV) in this experiment was −0.5–1.0 V vs the Ag/AgCl electrode. The experiment started with a 500-s open-circuit potential (OCP) measurement. This was followed by 10 CV scans at a scan rate of 0.10 V s^–1^ to stabilize the working electrode. Next, CV scans of the catalytic performance data were performed at a scan rate of 0.05 V s^–1^. Tafel plots were obtained by plotting the potential against the logarithm of the current density. The area used for calculating the current density includes both sides of the CP, that is, 0.88 cm^2^.

Electrochemical impedance spectroscopy (EIS) measurements were performed in the frequency range of 100 kHz to 10 mHz using a 10 mV AC perturbation amplitude at 0.35 V vs Ag/AgCl. The electrochemical behavior of ethanol oxidation was then interpreted using an equivalent circuit.

The ECSA serves as a pivotal parameter for appraising the number of electrochemically active sites within a catalyst and acts as a suitable benchmark for comparing different electrocatalysts.^25,26^ The ECSA for each electrode is derived from the electrochemical double-layer capacitance, which is determined by the linear response of non-Faraday capacitance current to the change of the scan rate, as illustrated by eq 1 and 2(27)
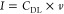
1
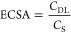
2where *I* represents the current, *v* the scan rate, and *C*_DL_ is the electrochemical double-layer capacitance. *C*_s_ represents the specific capacitance of the sample or the capacitance per unit area of the material on the atom-smooth planar surface under identical electrolyte conditions, and a *C*_s_ of Ni as 0.12 mF cm^–2^ was used.^28^ CV scans (−0.2–0 V vs Ag/AgCl) with scan rates of 20, 40, 60, 80 mV s^–1^ were performed to obtain the scan rate dependent current.

The number of electrons transferred during ethanol oxidation was tested by plotting the peak potential against the logarithm of scan rate and the peak current against the square root of scan rate using CVs at different scan rates (0.06–0.2 V s^–1^). The stability of the Ni/CP electrodes was investigated by 200 continuous CV scans at a scan rate of 0.10 V s^–1^, and the electrocatalytic data were collected every 20 scans. The stability of the electrode was evaluated by comparing the CV, EIS, and ECSA and reaction electron number before and after the stability test. Finally, the chronoamperometry (CA) test was completed at 0.72 V versus Ag/AgCl for 7200 s.

Potentials are IR-compensated and referenced to a reversible hydrogen electrode (RHE) via eq 3
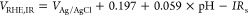
3where *V*_RHE,IR_ denotes the IR-compensated potential referenced to the RHE, *V*_Ag/AgCl_ represents the potential referenced to the Ag/AgCl electrode, *I* is the current, pH denotes the pH of the electrolyte, and *R*_s_ is the resistance of the circuit as determined by EIS measurements.

## 3 Results and Discussion

### 3.1 Structure of Ni/CP Electrodes

A series of Ni/CP electrodes were prepared with various deposition potentials. ICP-OES was used to determine the Ni concentration on the Ni/CP electrodes. The content of Ni increases with an increasing deposition potential. When the deposition potential increases from 1.35 to 1.55 V versus Ag/AgCl, the Ni content increases slightly from 0.010 to 0.012 mg_Ni_ cm^–2^, but as the deposition potential increases from 1.55 to 1.95 V versus Ag/AgCl, Ni content increases significantly from 0.012 to 0.330 mg_Ni_ cm^–2^.

**Figure 1:**
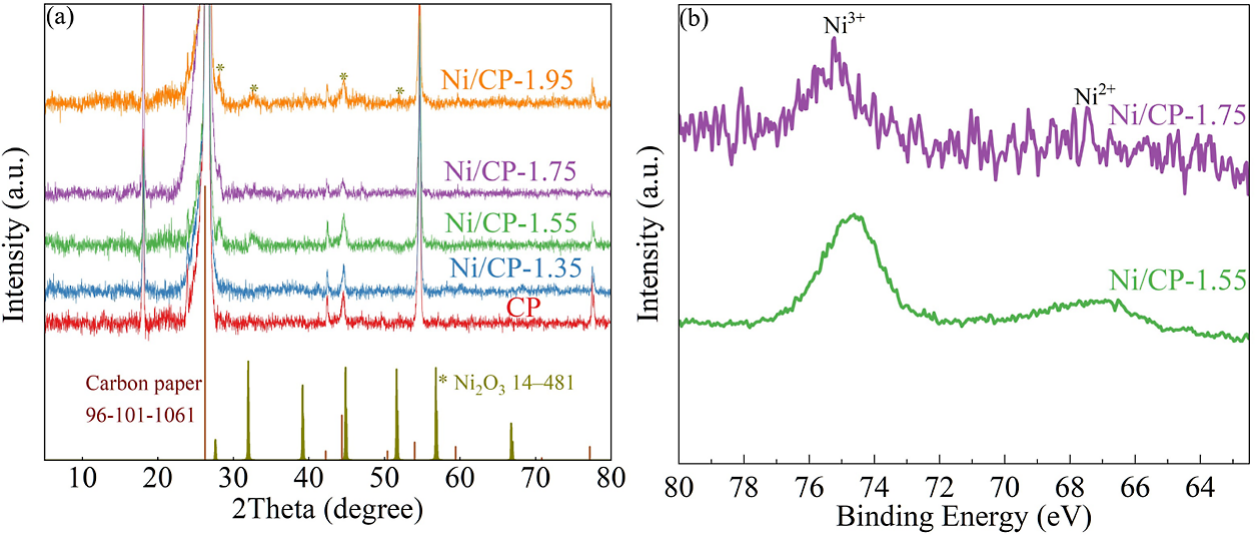
XRD patterns (a) and Ni 3p XPS spectra (b) of CP and electrodes prepared with various deposition potentials.

The structural characteristics of the Ni oxide/CP electrodes were examined using XRD. The XRD patterns (Figure 1a) of the obtained electrodes exhibit characteristic peaks at 26.23, 42.21, 44.37, 53.98, and 77.18°, aligning with the (002), (100), (101), (004), and (110) reflexes of CP, respectively. Notably, the Ni/CP-1.35 electrode, characterized by the lowest Ni loading and the absence of crystalline Ni oxides, closely resemble the XRD patterns of pristine CP. For Ni/CP-1.55, Ni/CP-1.75, and Ni/CP-1.95 electrodes with higher Ni loading, new peaks emerge at 27.6, 32.0, and 51.9°, which correspond to (101), (002), and (112) reflexes of Ni_2_O_3_, indicating the formation of Ni_2_O_3_.^29^

**Figure 2:**
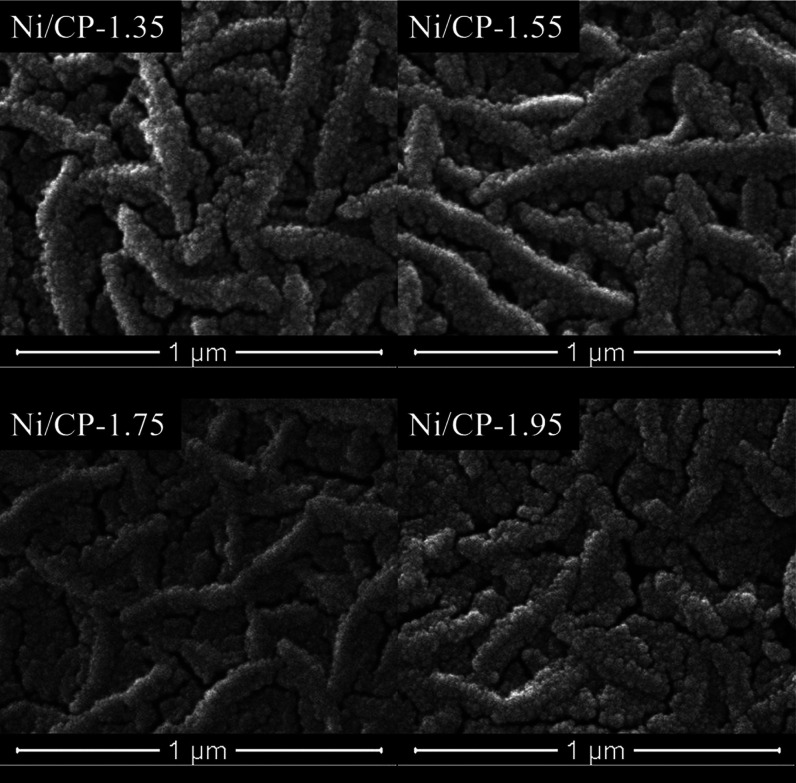
SEM images of Ni/CP electrode prepared with various deposition potentials. The scale bar is 1 μm.

To unravel the oxidation state of Ni species within the electrodes, we employed XPS analysis was employed. Given the challenging overlap between the Ni 2p peak and the F kll Auger peak derived from the CP,^30^ Ni 3p spectra were utilized for a more accurate assessment of the oxidation state. Figure 2b displays the Ni 3p spectra for the Ni/CP-1.55 and Ni/CP-1.75 electrodes. As can be seen, Ni/CP-1.55 and 1.75 electrodes show peaks at ∼67.1 and ∼74.6 eV, attributed to Ni^2+^ 3p 1/2 and Ni^3+^ 3p 3/2 orbital, respectively.^31^Figure S2 presents the O 1s spectra for the Ni/CP-1.55 and Ni/CP-1.75 electrodes. In the O 1s spectrum of Ni/CP-1.55, two principal peaks are observed at 531.9 and 529.6 eV, which are attributed to the C–O–C and C=O functional groups of the carbon paper, respectively. Meanwhile, the O 1s spectrum of Ni/CP-1.75 exhibits peaks at 533.1 and 531.2 eV, corresponding to the −O–C=O functional groups and Ni_2_O_3_.^32−34^ The predominate presence of Ni^3+^ again suggest the formation of Ni_2_O_3_. Although Ni typically oxidizes to form NiO, it has been reported that NiO can undergo further transformation to Ni_2_O_3_ through the following reaction, as described by Liu et al.^35^
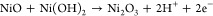


**Figure 3:**
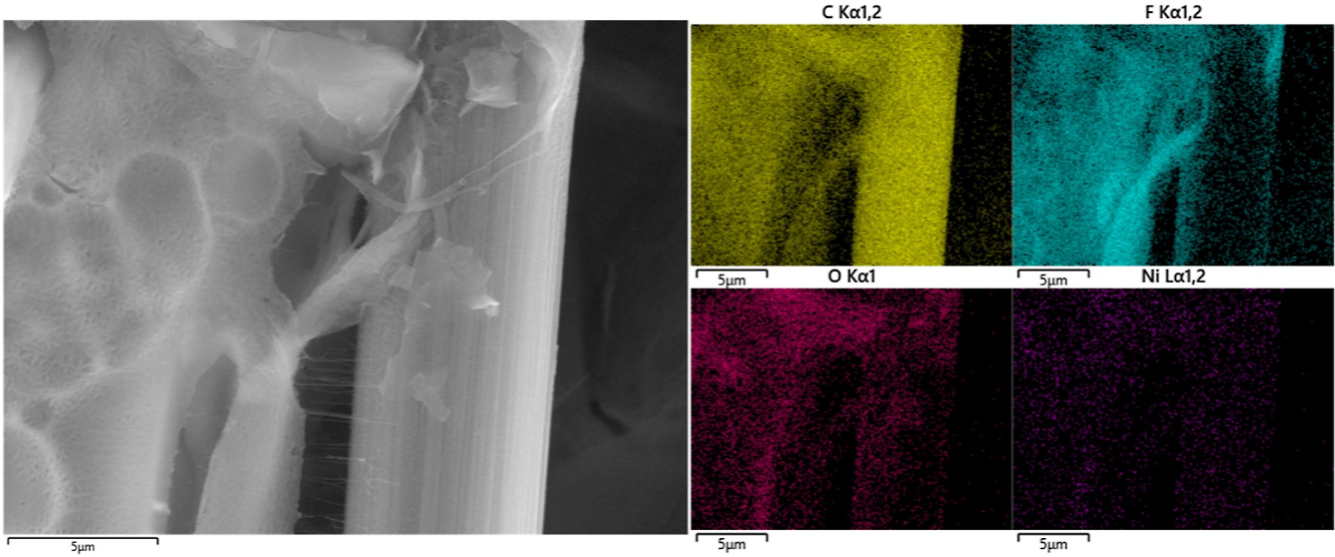
EDS element mapping of the Ni/CP-1.75 electrode.

The morphology of CP as well as the electrodes prepared using various deposition potentials were studied by SEM (Figures S3 and 2). The CP consists of a network of randomly oriented carbon fibers. Nickel oxide (Ni_2_O_3_) exists in the form of nanoplates, which aggregate into particles and adhere to the surface of carbon fibers. The EDS elemental mapping (Figure 3) corresponding to the Ni/CP-1.75 electrode provides additional insights, illustrating the uniform distribution of Ni across the CP substrate.

**Figure 4:**
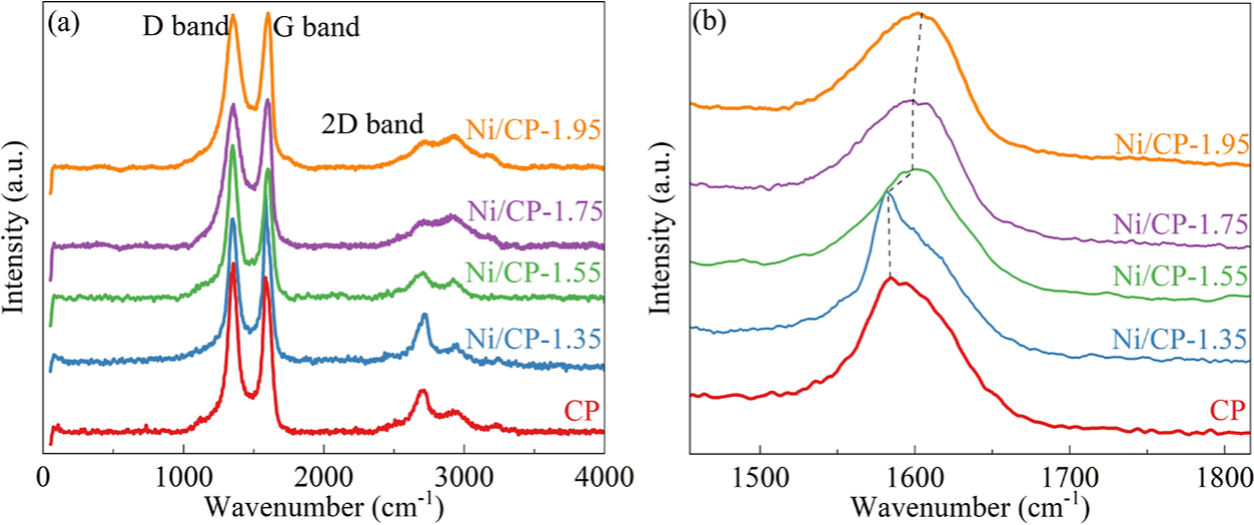
Raman spectra (a) with an expanded view of the G band region (b) for CP and electrodes prepared with various deposition potentials.

Figure 4 presents the Raman spectra of the electrodes prepared under different deposition potentials. Three distinct bands—D band (1350 cm^–1^), G band (1590 cm^–1^), and 2D band (2700 cm^–1^)—are evident. The D band is indicative of defects and lattice disorder within the carbon material’s structure. The G band, associated with sp^2^ carbon, is sensitive to external perturbations such as defects, doping, strain, and temperature.^36^ As shown in Figure 4b, there is a significant blue shift of the G band with an increasing deposition potential. This shift indicates that the CP was doped with a P-type semiconductor,^37^ which is Ni_2_O_3_ in this case.^38−40^ This again suggests the successful preparation of the Ni_2_O_3_/CP electrode with various doping degree.

The 2D bands are sensitive to the presence of free electrons or free holes in the semiconductors doped into the carbon material, and in particular, the Full width at half-maximum (fwhm) of the 2D bands of graphene-based carbon materials is proportional to the Fermi level, and a bigger fwhm of the 2D peaks indicates enhanced ability in charge exchange and lower charge transfer energy barriers, favoring redox reactions.^41^ The corresponding 2D fwhm values for Ni/CP-1.35, Ni/CP-1.55, Ni/CP-1.75, and Ni/CP-1.95 are 125.40, 201.15, 210.80, and 238.00 cm^–1^, respectively. The observed increase in the fwhm of the 2D band with rising potential correlates with the shift in the position of the G band position, suggesting a stronger interaction between Ni_2_O_3_ and CP.

### 3.2 Electrochemical Oxidation of Ethanol

**Figure 5:**
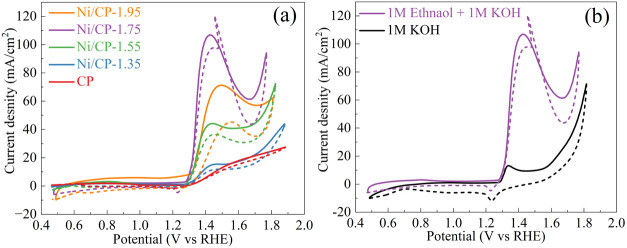
(a) CV curves over CP and Ni/CP electrodes prepared using various deposition potentials measured in 1 M KOH + 1 M ethanol; (b) CV curves over Ni/CP-1.75 electrode measured in 1 M KOH + 1 M ethanol and 1 M KOH, respectively. Scan rate: 50 mV/s; IR compensated. The solid curves represent the forward scan, and the dashed curve indicates the backward scan.

The electrocatalytic activity of CP and the Ni/CP electrodes for ethanol oxidation was systematically investigated using a three-electrode system in 1.0 M KOH and 1.0 M ethanol electrolyte. The CV curves are presented in Figure 5, showing 2 redox peaks.

For instance, the CV curve of the Ni/CP-1.75 catalyst exhibits an oxidation peak near 1.417 V vs RHE in the forward scan and a spike near 1.454 V versus RHE in the reverse scan. The oxidation peak at 1.417 V versus RHE is attributed to ethanol oxidation, while the appearance of the spike at 1.454 V versus RHE in the reverse scan is attributed to the presence of incompletely oxidized carbonaceous species. This species is removed by oxidation during the reverse scan process, leading to the rerelease of surface active sites. Similar behavior has been reported in the literature.^26,42^ The decrease in current density observed at elevated potentials (greater than 1.45 V vs RHE) can be ascribed to the further oxidation of Ni (III) to a higher oxidation state, resulting in the formation of an inactive oxide layer.^43^ This layer of inactive oxides could potentially impede the ethanol oxidation process by forming an obstructive coating on the catalyst’s surface.^44^

Moreover, it observed that the electrode activity for ethanol oxidation correlates well with the deposition potentials. While the CP and the Ni/CP electrodes exhibit close onset potentials (forward scan) at 1.29–1.31 V versus RHE, their peak potentials (forward scan) and current densities (forward scan) show obvious difference. Compared with CP, Ni/CP-1.35 shows only slight enhancement for electrochemical ethanol oxidation. However, with higher deposition potentials, a significant enhancement is observed. With increasing deposition potentials, the peak current densities first increase from 14.8 mA cm^–2^ (Ni/CP-1.35) to a maximum of 106.9 mA cm^–2^ over Ni/CP-1.75, then decreases to 71.2 mA cm^–2^ with further increasing deposition potential (Ni/CP-1.95). Furthermore, we compared the Ni/CP-1.75 catalyst’s peak current density for ethanol oxidation in alkaline electrolyte with state-of-the-art nickel-based catalysts as detailed in Table S1. It is revealed that the Ni_2_O_3_/CP catalyst exhibits a peak current density surpassing those of state-of-the-art nickel-based catalysts, which signifies its superior catalytic efficacy for ethanol oxidation.

The catalytic activity of Ni/CP-1.75 in a 1 M KOH solution was also investigated (Figure 5b). The onset potential is shifted 1.6 V versus RHE, indicating the high current density in ethanol containing electrolyte is ascribed to the oxidation of ethanol. Moreover, redox couple peaks at 1.33 and 1.23 V versus RHE, ascribed to Ni^2+^/Ni^3+^,^45^ are observed. Though the reduction peak is also observed during ethanol oxidation, the oxidation peak disappears due to overlap with ethanol oxidation. We have also deposited Ni on various substrates (Fe foam, Ni foam, and Ti foam) under uniform conditions to assess their suitability for ethanol oxidation (Figure S4). The observed low activity of nickel on Fe, Ni, and Ti foams indicates that CP is a superior substrate for Ni electrodes.

**Figure 6:**
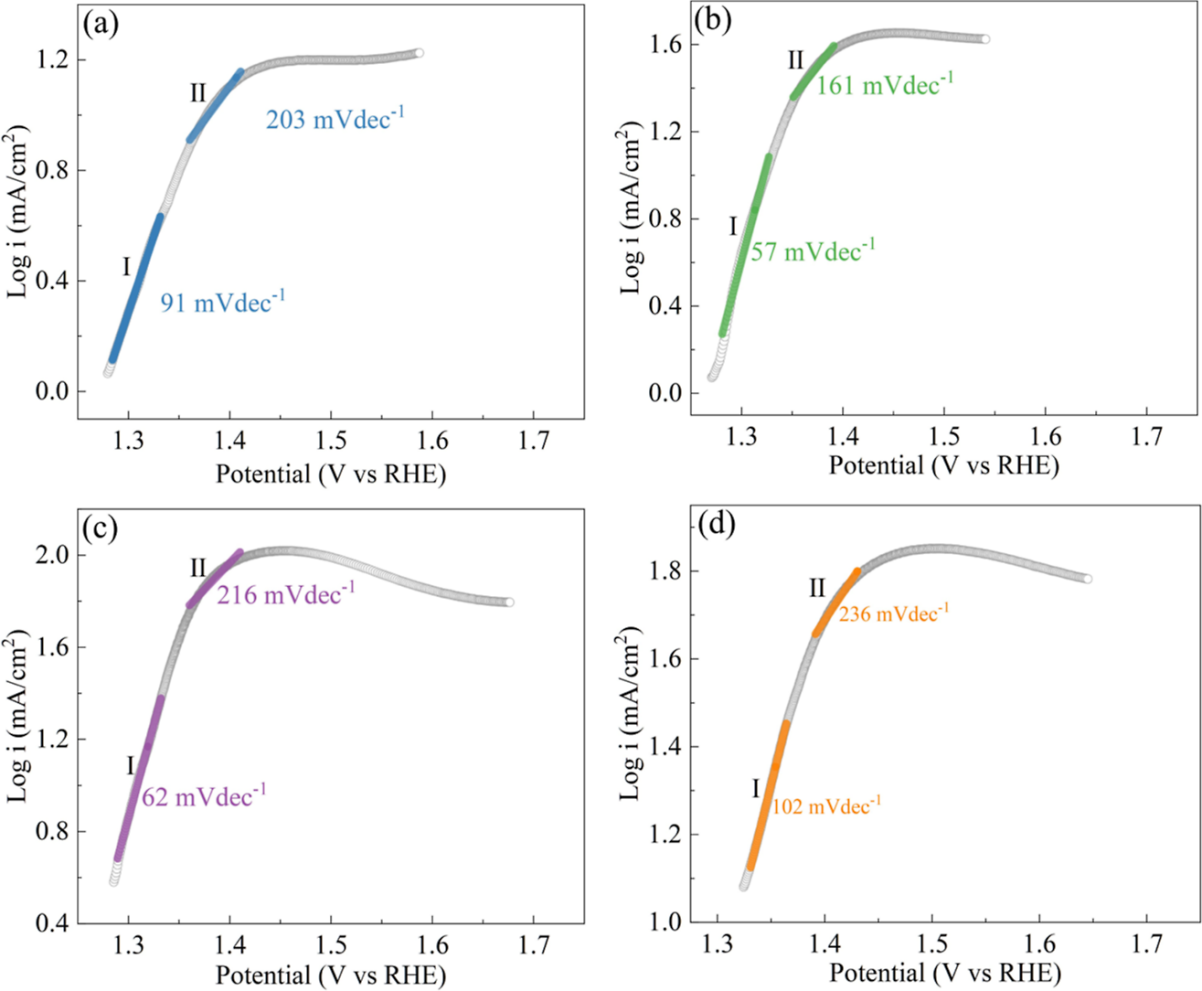
Tafel plots of ethanol oxidation over (a) Ni/CP-1.35, (b) Ni/CP-1.55, (c) Ni/CP-1.75, and (d) Ni/CP-1.95. Scan rate: 50 mV s^–1^; IR compensated; electrolyte: 1 M ethanol and 1 M KOH solution.

The Tafel slope, an intrinsic characteristic of electrocatalysts, underwent a thorough investigation to assess the impact of various deposition potentials on the kinetics of ethanol oxidation. The Tafel plots in Figure 6 reveal two distinct linear regions. In the low potential range (1.29–1.33 V vs RHE), the Tafel slope locates between 57–102 mV dec^–1^, indicating the adsorption of hydroxyl group on catalyst’s surface determines the ethanol oxidation kinetics.^46^ In the high potential range (1.36–1.41 V vs RHE) the Tafel slopes increases significantly to 161–236 mV dec^–1^. This escalation signifies is attributed to the emergence of an inactive oxide layer on catalyst’s surface, impacting the kinetics and resulting in the heightened Tafel slope.^44,46^ Notably, Ni/CP-1.55 and Ni/CP-1.75 exhibit smaller Tafel slopes compared to Ni/CP-1.35 and Ni/CP-1.95. This indicates faster electron transfer kinetics—a feature of high-performance electrocatalysts.^47^ Consistent with this, the Ni/CP-1.75 catalyst demonstrated high catalytic activity for ethanol oxidation.

**Figure 7:**
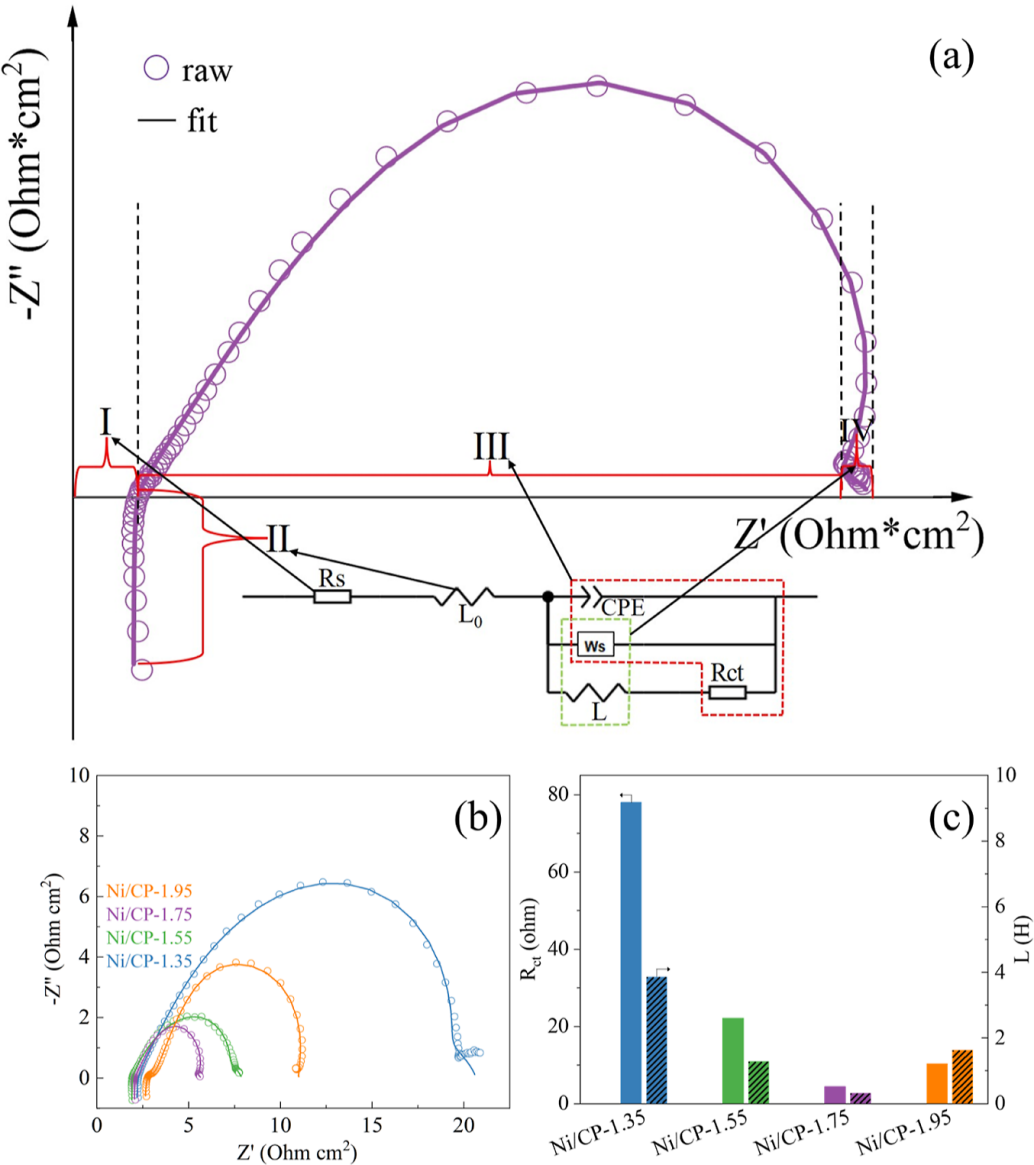
(a) The Nyquist plot of the Ni/CP-1.75 electrode together with an equivalent circuit. (b) Nyquist plots with fit curves of Ni/CP electrodes deposited at various potentials. (c) *R*_ct_ and *L* of the Ni/CP electrodes deposited at various potentials. Measured at 0.35 V vs Ag/AgCl with an amplitude of 10 mV from 100 kHz to 10 mHz in 1 M ethanol and 1 M KOH solution.

EIS measurements were conducted in a 1 M KOH and 1 M ethanol solution, employing a potential of 0.35 V vs Ag/AgCl. The Nyquist plots together with an equivalent circuit used for fitting are illustrated in Figure 7a. The Nyquist plots of the electrodes can be divided into four electrochemically relevant parts, corresponding to an equivalent circuit diagram (Figure 7a). The first part (I) of the Nyquist diagram is indicated by the intersection of the impedance data with the horizontal axis at a nonzero value. In this part, *R*_s_ represents the sum of the resistances of the electrolyte, wire, and catalyst. The second part (II) is shown by the impedance data on the negative half axis of the longitudinal axis at high frequencies. Here, *L*_0_ represents the intrinsic inductance of the wire coils, which is related to the induced reactance of the metal in the wire.^48^ The third part (III) is characterized by an irregular semicircle, representing the electrical response of the double-layer capacitance on the surface of the working electrode, the charge transfer resistance, and the reaction-related diffusion or mass transfer impedance. These are denoted by CPE, *R*_ct_, and *W*_s_, respectively.^49^ The fourth part (IV) at the low-frequency region shows a straight line-like portion, resulting from the combined effect of the apparent inductance affected by the adsorption of reactants on the surface of the electrode and the diffusion or mass transfer impedance associated with the reaction. These are denoted by *L* and *W*_s_, respectively.^50,51^

Figure 7b shows the Nyquist plots of Ni/CP electrodes deposited at different potentials along with the fitted curves using the equivalent circuit of Figure 7a. The obtained *R*_ct_ and *L* value are presented in Figure 7c. *R*_ct_ shows an order of Ni/CP-1.75 (4.43 Ω·cm^2^) < Ni/CP-1.95 (10.34 Ω·cm^2^) < Ni/CP-1.55 (22.13 Ω·cm^2^) < Ni/CP-1.35 (77.99 Ω·cm^2^), while the L shows an order of Ni/CP-1.75 (0.32 H) < Ni/CP-1.55 (1.28 H) < Ni/CP-1.95 (1.63 H) < Ni/CP-1.35 (3.86 H). Smaller L implies reduced inductive resistance due to reactant adsorption on the catalyst surface, while smaller *R*_ct_ indicates decreased charge transfer resistance. Both facilitate faster electrochemical ethanol oxidation. Accordingly, lower *L* and *R*_ct_ contribute to a higher peak current density. The lower *R*_ct_ value for Ni/CP-1.75 is potentially associated with the proper loading of Ni and Ni_2_O_3_–CP interactions, as suggested by Raman spectroscopy. However, excessive deposition of Ni_2_O_3_ on CP in Ni/CP-1.95 cannot lead to further increasing amount of P-type carriers, meanwhile accumulation of the less conductive Ni_2_O_3_ on the surface of CP may inhibit ethanol oxidation.

**Figure 8:**
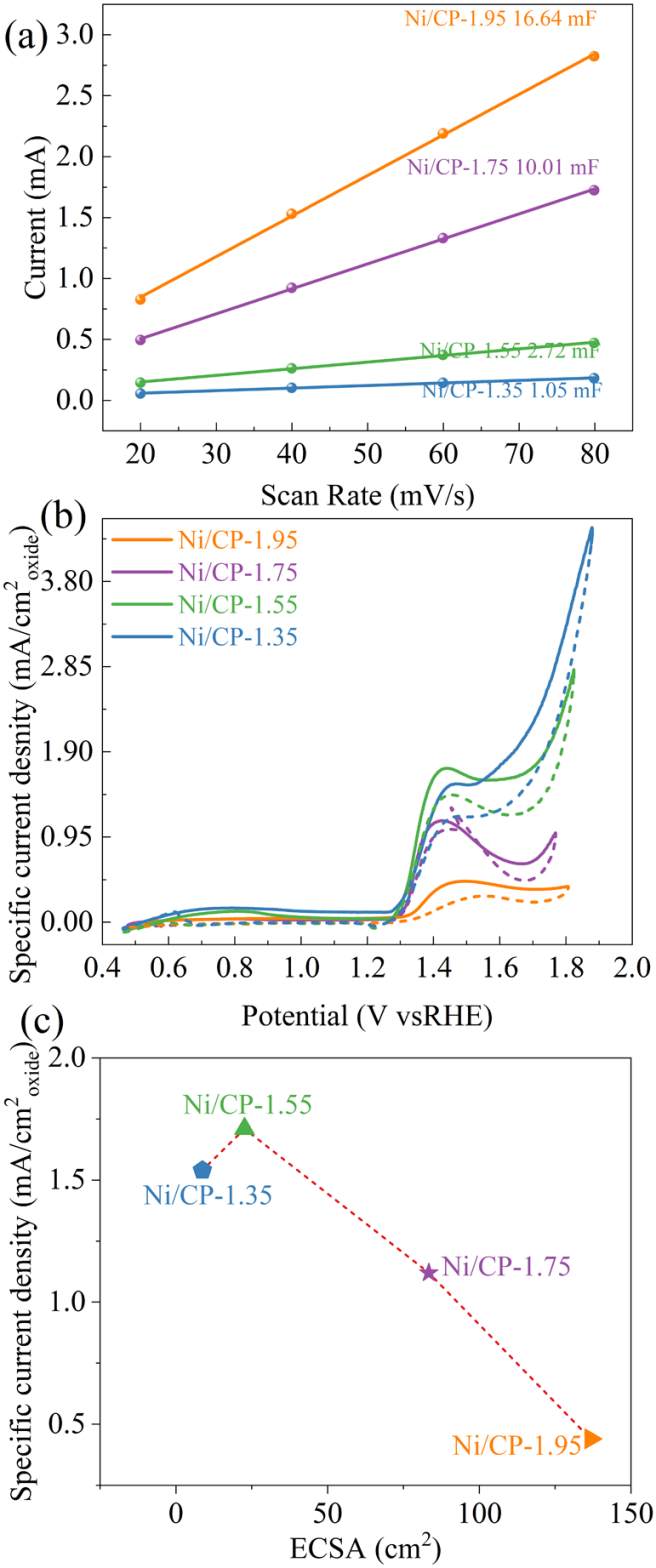
(a) Scan rate dependence of the current for Ni/CP-1.35, Ni/CP-1.55, Ni/CP-1.75, and Ni/CP-1.95; (b) ECSA based CV curves for Ni/CP-1.35, Ni/CP-1.55, Ni/CP-1.75, and Ni/CP-1.95 in 1 M KOH + 1 M Ethanol solution at the 50 mV s^–1^; (c) correlation between specific peak current density and ECSA.

The electrochemical double layer capacitances (*C*_dl_) for Ni-1.35 V/CP, Ni/CP-1.55, Ni/CP-1.75, and Ni/CP-1.95 are 1.05, 2.72, 10.01, and 16.64 mF, respectively (Figure 8a). This is consistent with the increasing Ni loading as the deposition potential increases. Notably, Ni/CP-1.95 exhibits the largest *C*_dl_ but falls short of the catalytic activity observed for Ni/CP-1.75, suggesting that even though Ni/CP-1.95 possesses more active sites, the excessive Ni_2_O_3_ deposition leads to diminished intrinsic activity. To further prove this, the ECSA was also calculated to obtain the ECSA based CV curves, a depiction of the intrinsic electrocatalytic activity. The ECSA for Ni-1.35 V/CP, Ni/CP-1.55, Ni/CP-1.75, and Ni/CP-1.95 are 8.75, 22.63, 83.38, and 136.67 cm^2^, respectively. The ECSA based CV curves show that with increasing deposition potential, the specific peak current density (Figure 8b, forward scan) increases first and then decreases, peaking at a deposition potential of 1.55 V versus Ag/AgCl. This would be attributed to the proper amount of Ni_2_O_3_ deposited on CP, forming favorable Ni_2_O_3_–CP interactions and accelerating ethanol oxidation. The specific peak current density is also correlated with the ECSA. As can be seen, with increasing ECSA, the specific peak current density increases first then decreases. Ni/CP-1.75 possesses both relatively high specific current density and ECSA, achieving the highest apparent activity for ethanol oxidation. We also normalized the current density relative to the Ni mass, as determined by ICP-OES, and depicted the Ni-mass-normalized CV curves in Figure S5. The peak current densities for the catalysts Ni/CP-1.35, Ni/CP-1.55, Ni/CP-1.75, and Ni/CP-1.95 are found to be 1.30, 3.23, 1.38, and 0.19 A mg_Ni_^–1^, respectively. Notably, the highest nickel mass-based current density is obtained by Ni/CP-1.55, aligning with the ECSA normalized results.

**Figure 9:**
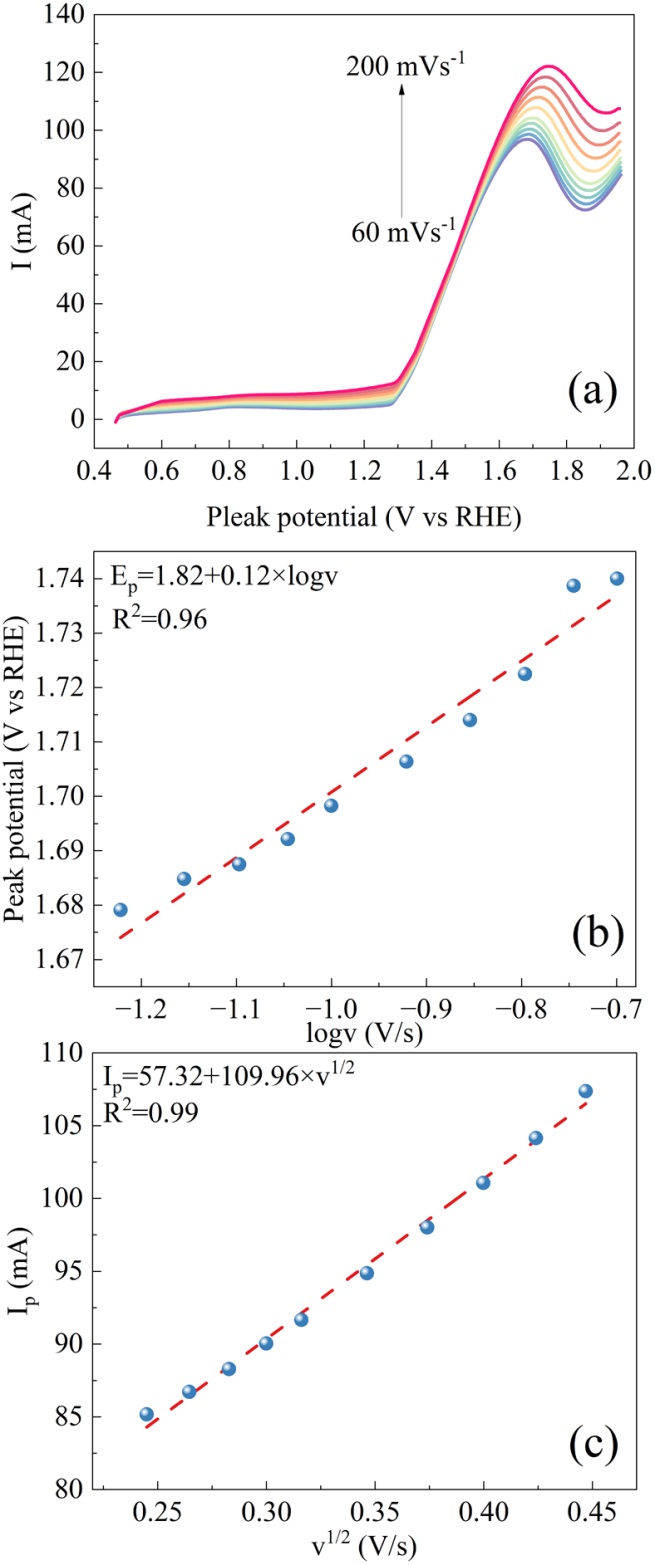
(a) Forward scans of CV curves (forward scan) obtained over Ni/CP-1.75 in 1 M KOH + 1 M ethanol with various scan rates of 60, 70, 80, 90, 100, 120, 140, 160, 180, and 200 mV s^–1^; (b) dependence of the peak potential (*E*_p_) on the logarithm of the scan rate, log*vv*, with linear fitting results; (c) dependence of the peak current (*I*_p_) on the square root of the scan rate (*v*^1/2^) with linear fitting results.

To shed light on the ethanol oxidation mechanism, CV measurements were performed at varying scan rates (60–200 mV s^–^) over Ni/CP-1.75 in a 1 M KOH + 1 M ethanol solution. Figure 9 illustrates the CV curves (forward scan) as well as *E*_p_–log *v* and *I*_p_–*v*^1/2^ plots, together with linear fitting results. Notably, the shift of the peak to higher potential as the scan rate increases signifies the irreversibility of the ethanol oxidation process. Moreover, the linear relationship between *E*_p_ and log *v*, together with that between *I*_p_ and *v*^1/2^, as expressed by eqs 3 and 4, indicates a completely irreversible diffusion process.^52,53^
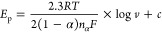
4
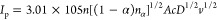
5where *R* is the gas constant (8.314 J K^–1^·mol^–1^), *T* is the temperature (298.18 K), α is the electron transfer coefficient, *n*_α_ is the number of electrons transferred in the rate-determining step, *F* is Faraday constant (96485 Pa·m^3^ K^–1^ mol^–1^), *n* is the total number of electrons transferred during the reaction, *A* is the ECSA (83.38 cm^2^), *c* is the concentration of reactants (0.001 mol cm^–3^), *D* is the diffusion coefficient (1.23 × 10^–5^ cm^2^ s^–1^^54^), and *v* is the scan rate (V s^–1^). Using the slope of the *E*_p_–log *v* plot, the (1 – α)*n*_*α*_ is determined to be 0.25. Moreover, with the slope of the *I*_p_–*v*^1/2^ plot, *n* is determined to be 2.5, suggesting that ethanol undergoes oxidation on the catalyst’s surface involving the transfer of 2.5 electrons and forming acetic acid.

The oxidation of ethanol to acetic acid over Ni-based catalysts have been studied by Fleischmann et al.,^55−57^ proposing the following mechanism, eqs 1–5.
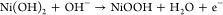
1a
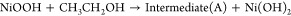
2a
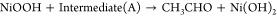
3a
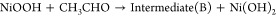
4a
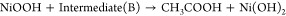
5a

Electrochemical oxidation of ethanol over Ni-based catalysts is mainly dependent on the reversible redox transformations between Ni (II) and Ni (III). Meanwhile, in the presence of an alkaline electrolyte, Ni species are shown to form an insoluble adherent hydroxide layer.^18^ It can be speculated that the oxidation of ethanol over Ni_2_O_3_/CP initiates with the electro-oxidation of Ni (II) to Ni (III), which corresponds to the observed small oxidation peak in the KOH electrolyte. Subsequently, ethanol is oxidized by Ni (III), forming acetaldehyde and acetic acid and converting Ni (III) back to Ni (II). The presence of Ni (III) in the catalysts may favor the formation of NiOOH, promoting ethanol oxidation.

**Figure 10:**
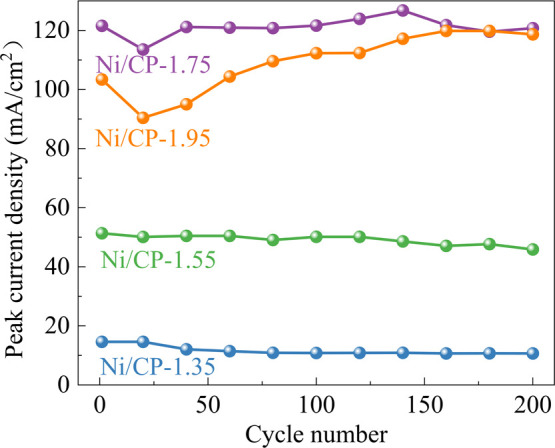
Evolution of peak current densities (forward scan) with an increasing number of cycles. Scan range: 0.46–1.96 V vs RHE; scan rate: 100 mV s^–1^; IR compensated; electrolyte: 1 M ethanol and 1 M KOH solution.

To assess the stability of the electrodes for ethanol oxidation, 200 CV scans were performed with a scan rate of 100 mVs^–1^ in 1 M KOH and 1 M ethanol solution. Figure 10 depicts the evolution of the peak current density. Both Ni/CP-1.35 and Ni/CP-1.55 catalysts exhibits stable peak current densities with increasing number of cycles, highlighting the robust stability of theses electrodes. Conversely, the peak current densities of Ni/CP-1.75 and Ni/CP-1.95 electrodes experience a significant decrease in less than 20 cycles. Beyond 20 cycles, the peak current density gradually increases. Notably, after 160 cycles, the peak current density of the Ni/CP-1.95 electrode is close to that of Ni/CP-1.75.

**Figure 11:**
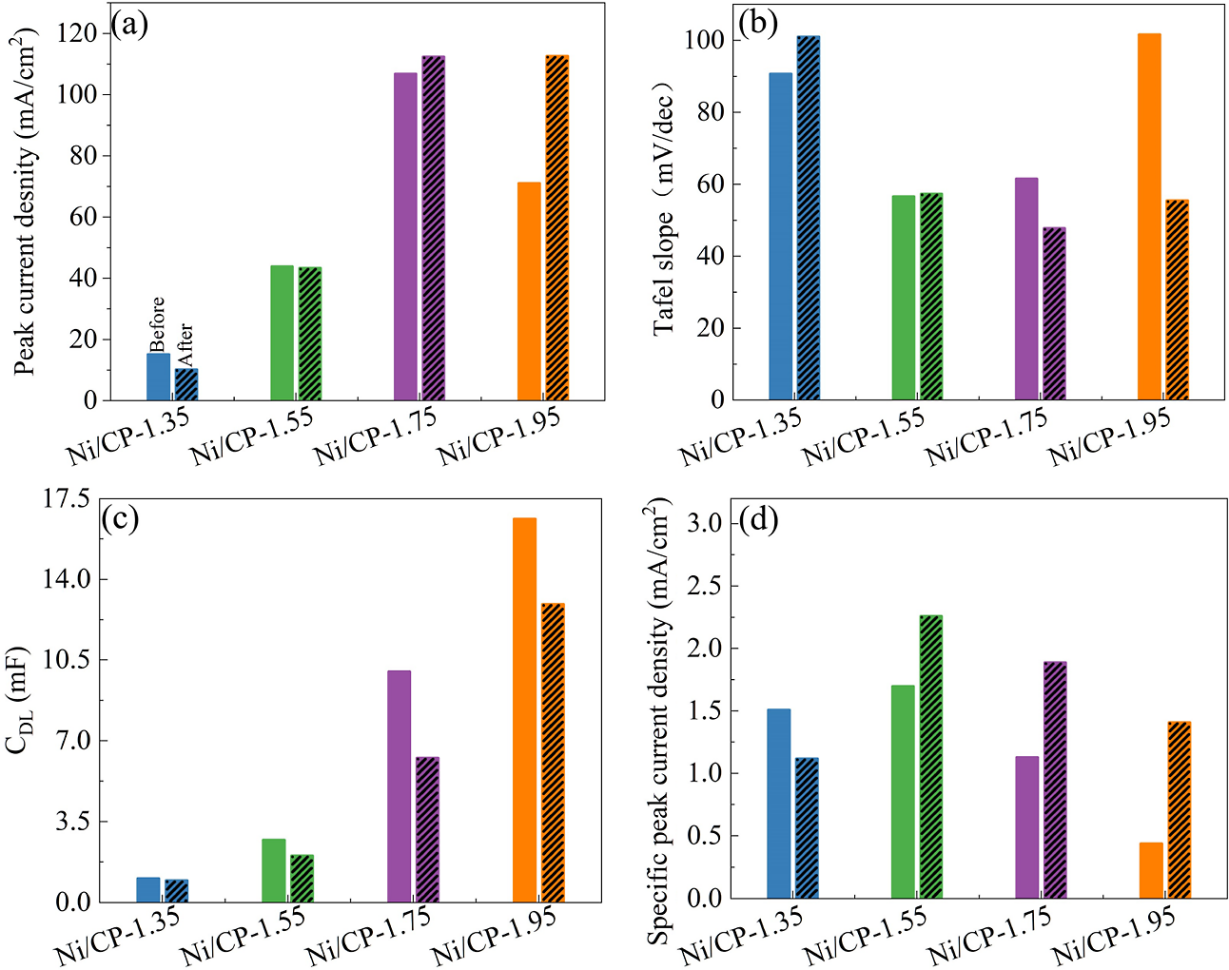
Comparison of the peak current density (a), Tafel slope (b), *C*_dl_ (c), and specific peak current density (d) of various electrodes before and after stability test, 200 CV scans, 0.46–1.96 V vs RHE, 100 mV s^–1^; IR compensated; electrolyte: 1 M ethanol and 1 M KOH solution.

The CV curves, Tafel plots, *C*_dl_, and specific peak current density of various electrodes before and after stability test was also measured (Figures S6–10). Figure 11 compares the peak current density (forward scan), Tafel slope (in range I), *C*_dl_, and the specific peak current density. The peak current density of Ni/CP-1.35, Ni/CP-1.55, and Ni/CP-1.75 shows only slight change, while that of Ni/CP-1.95 increases significantly from 71.2 to 112.7 mA cm^–2^ (Figure 11a), consisting with a significant decrease of Tafel slope from 102 to 56 mv dec^–1^ (Figure 11b). A decrease in the *C*_dl_ for all the electrodes is observed (Figure 11c), while the specific peak current density show an obvious increase except for Ni/CP-1.35 (Figure 11d). This indicates that the reconstruction of catalysts’ structure during ethanol oxidation varies both the ECSA and catalysts’ intrinsic activity. While a trade-off between them occurs, the apparent current density of Ni/CP-1.35, Ni/CP-1.55, and Ni/CP-1.75 change only slightly. The increased specific current density is also partially ascribed to the change of reaction due to the accumulation of the intermediate, which is supported by the number of electrons transferred during reaction change from 2.5 to 5.9 after stability test (Figure S11). However, for Ni/CP-1.95, the *C*_dl_ only reduces slightly, while a significant increase of the specific current density appears, thus leading to significantly improved apparent current density.

**Figure 12:**
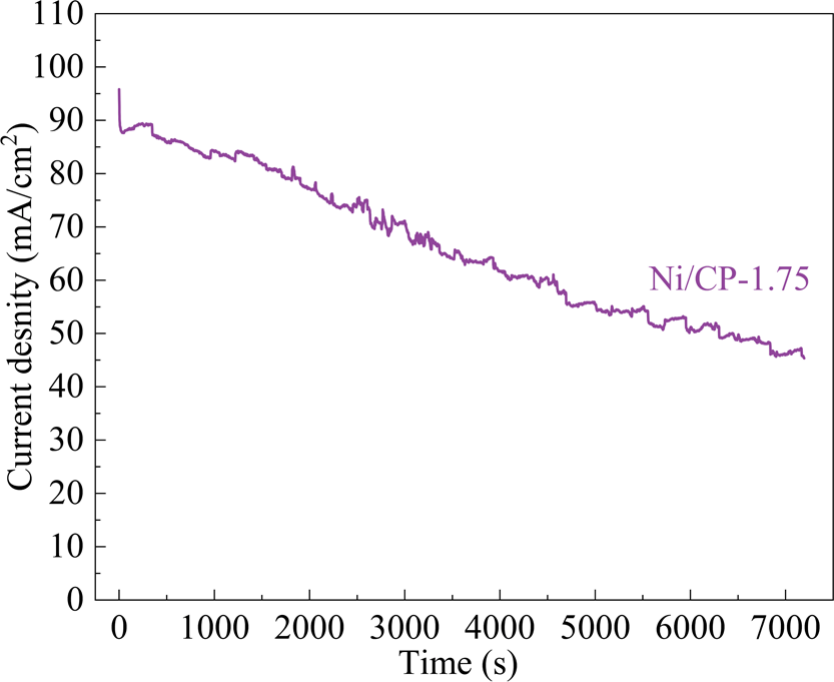
Chronoamperometric responses of Ni/CP-1.75 catalyst in 1 M ethanol +1 M KOH solution at 1.68 V vs RHE.

Chronoamperometry was employed to investigate the stability of the Ni/CP-1.75 electrode (Figure 12). Notably, the current density exhibits significant decay during the initial 50 s of the test. Subsequently, at 7200 s, the current density decreases by 48%. This decline in current density could be attributed to the adsorption of incomplete oxidation products on the catalyst surface or the rapid depletion of ethanol concentration near the electrode–solution interface.^23,58,59^ However, during CV measurements (Figure 10), the electrode demonstrates stability. This stability may be due to the CV process removing carbonaceous material and releasing surface-adsorbed species, thereby maintaining a higher current density.^26,42^

The post reaction Ni/CP-1.75 electrode was analyzed by XRD to analyze the change of nickel’s oxidation state (Figure S12). Diffraction reflexes of Ni_2_O_3_ still exist. Moreover, New peaks emerged at 30.1, 32.7, 34.5, and 40.6°, corresponding to the (010), (110), (013), and (210) facets of Ni(OH)_2_. The appearance of Ni(OH)_2_ can be ascribed to the conversion of NiO into crystalline Ni(OH)_2_ on the catalyst’s surface within an alkaline environment.^18^

## 4 Conclusions

Our investigation into CP-supported Ni_2_O_3_ as an electrocatalyst for ethanol oxidation has yielded significant insights into the effective design and optimization of catalysts for renewable energy applications. By employing electrodeposition, we successfully deposited Ni_2_O_3_ onto the CP, creating a highly effective catalyst for ethanol oxidation. The key to our catalyst’s high performance lies in the tailored doping degree, optimized through the control of electrodeposition potential. This optimization leads to a catalyst that exhibits both high intrinsic activity and a large ECSA, which are crucial for efficient electrocatalysis.

The resulting catalyst demonstrates a high performance for oxidizing ethanol to formic acid coupled with commendable stability, underscoring its practical application potential. The interaction/interface between Ni_2_O_3_ and CP plays a pivotal role in this enhanced performance, indicating the importance of interface engineering in catalyst design. This study not only advances our understanding of Ni-based catalysts for ethanol oxidation but also contributes to the broader field of electrocatalysis, opening new avenues for the development of renewable energy technologies.

## Supporting Information Available

Deposition current density profile, O 1s XPS spectra, SEM image of carbon paper, CV curves for Ni based catalysts supported on various materials, CV curves normalized by Ni mass, CV curves after stability test, Tafel plots after stability test, scan rate dependence of the current after stability test, ECSA based CV curves after stability test, Nyquist plots after stability test, determination of the number of electrons transfer in reaction after stability test, and XRD patterns after stability test. Comparison of peak current density with state of art Ni-based catalysts (PDF)
