# Supplementary material for: Optimized Electrodeposition of Ni2O3 on Carbon Paper for Enhanced Electrocatalytic Oxidation of Ethanol
Source: ACS Omega. 2024 Jun 29;9(28):30404–14. doi: 10.1021/acsomega.4c01658 (PMC11256107; doi:10.1021/acsomega.4c01658)
Supplement: Supplementary file 1 — ao4c01658_si_001.pdf [file ao4c01658_si_001.pdf]

Optimized Electrodeposition of Ni_2O_3 on Carbon Paper for Enhanced Electrocatalytic Oxidation of Ethanol

Ruixing Du,[a] Qitong Zhong,[a] Xing Tan,[a] Longfei Liao,[b] Zhenchen Tang,[a] Shiming Chen,[c] Dafeng Yan,[d] Feng Zeng[a,*]

[a] State Key Laboratory of Materials-Oriented Chemical Engineering, College of Chemical Engineering, Nanjing Tech University, Nanjing 211816, Jiangsu, China

[b] School of Materials Science and Engineering, Harbin Institute of Technology (Shenzhen), Shenzhen 518055, Guangdong, China

[c] School of Intelligent Medicine, China Medical University, Shenyang 110122, Liaoning, China

[d] College of Chemistry and Chemical Engineering, Hubei University, Wuhan 430062, China

* Corresponding author: zeng@njtech.edu.cn

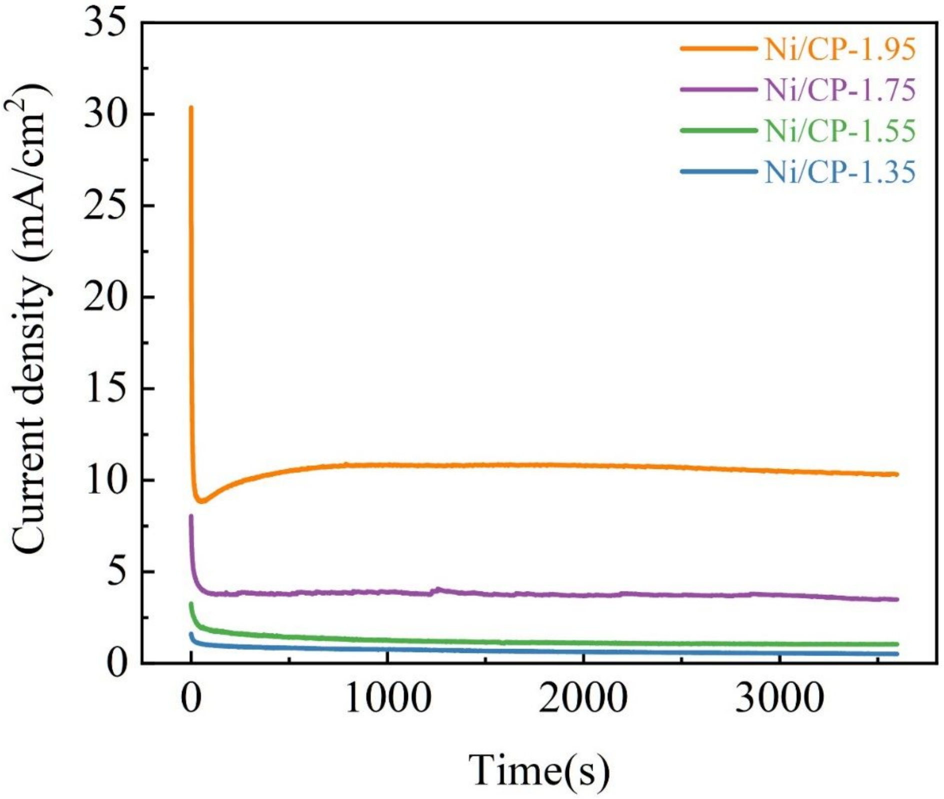

Figure S1. Deposition current density profile for Ni/CP-1.35, Ni/CP-1.55, Ni/CP-1.75, and Ni/CP-1.95.

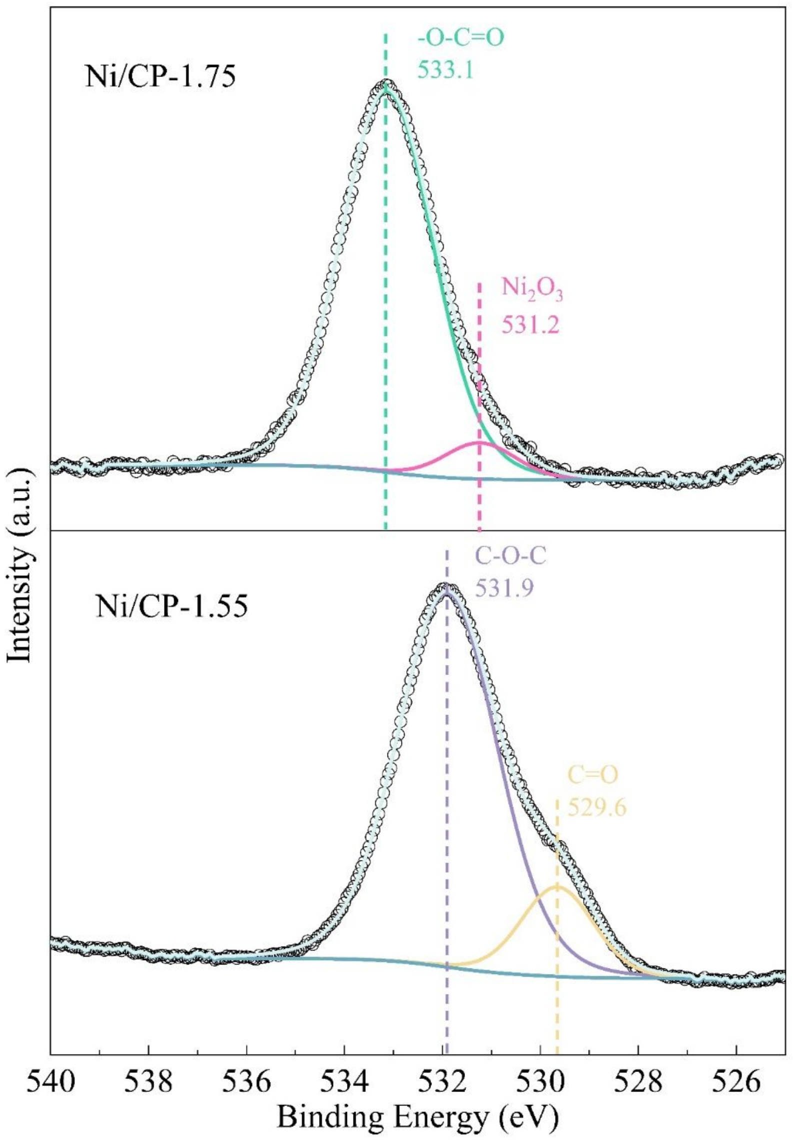

Figure S2. O 1s XPS spectra of electrodes prepared various deposition potentials.

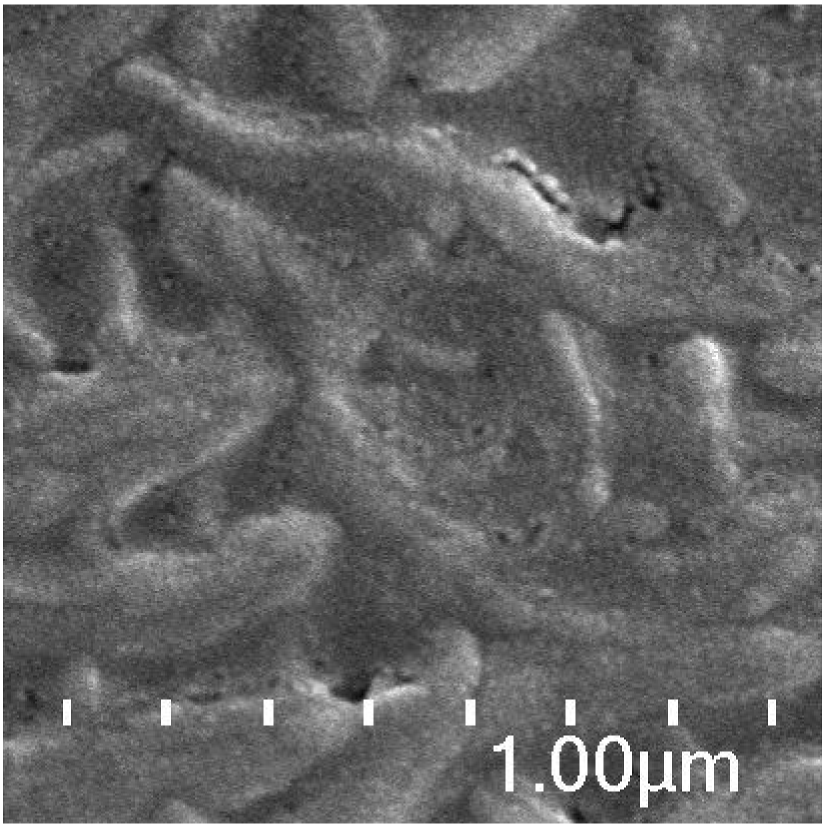

Figure S3. SEM image of carbon paper.

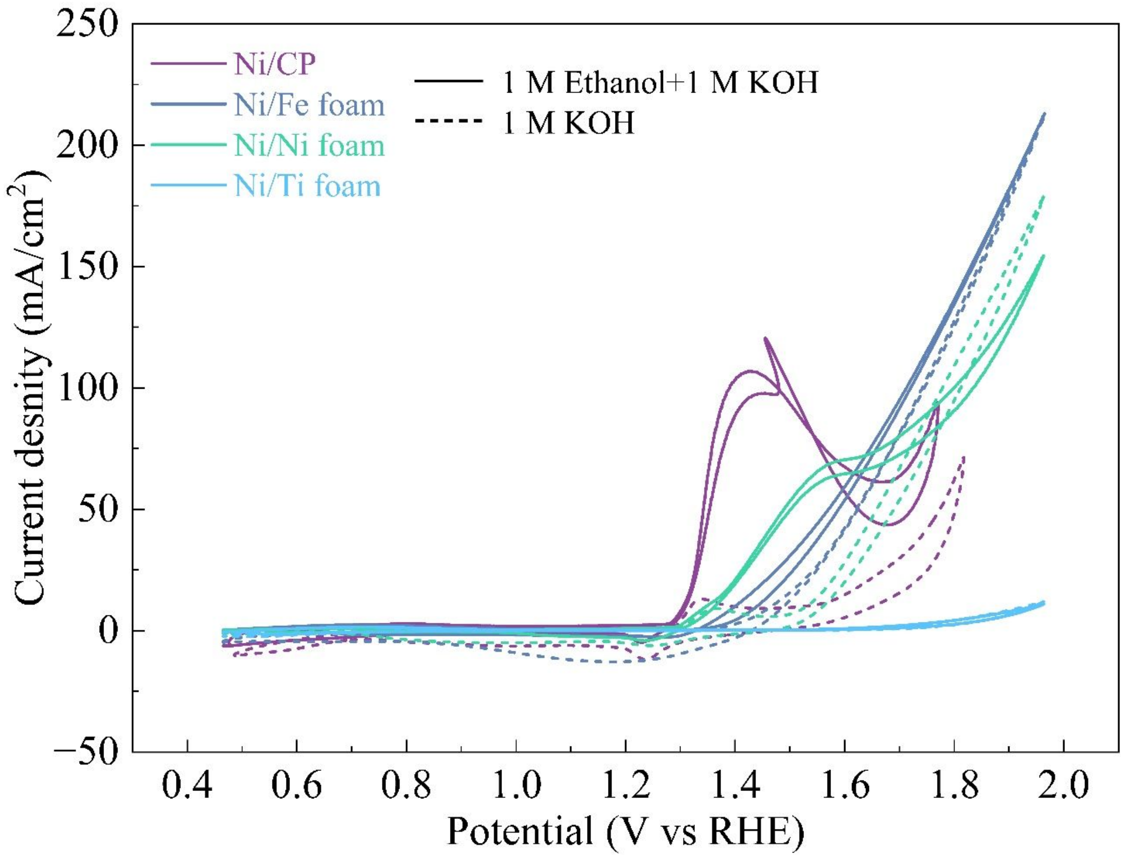

Figure S4. The CV curves for Ni based catalysts supported on various materials. Scan rate: 50 mV/s; electrolyte: 1 ethanol and 1 M KOH or 1 M KOH; IR compensated. The solid curves represent the forward scan and the dash curve indicate the backward scan.

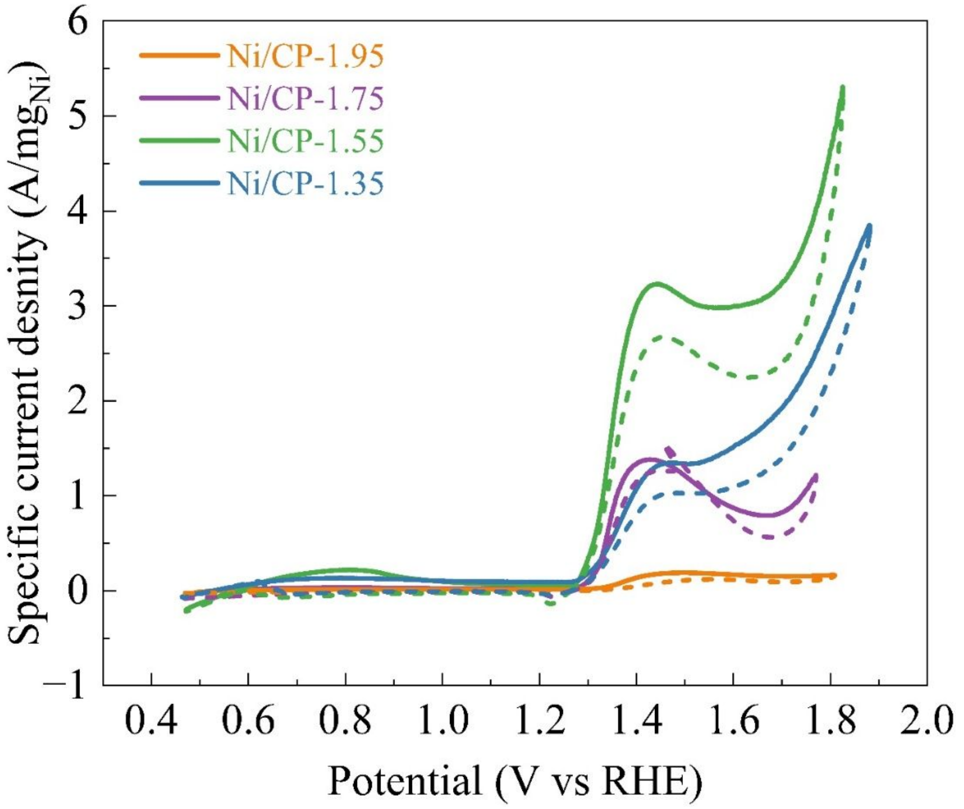

Figure S5. CV curves normalized by Ni mass for Ni/CP-1.35, Ni/CP-1.55, Ni/CP-1.75, Ni/CP-1.95. Scan rate: 50 mV/s; electrolyte: 1 ethanol and 1 M KOH; IR compensated. The solid curves represent the forward scan and the dash curve indicate the backward scan.

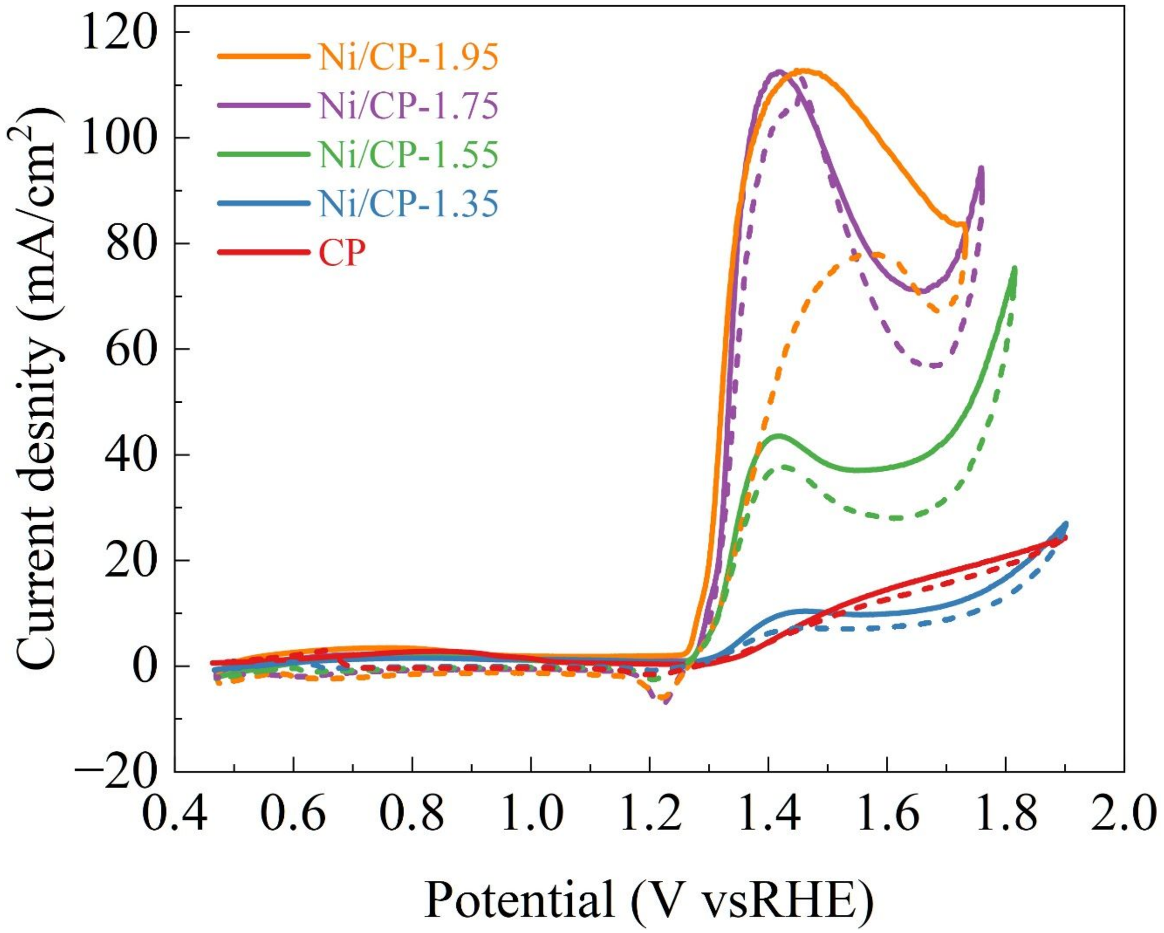

Figure S6. CV curves of CP, Ni/CP-1.35, Ni/CP-1.55, Ni/CP-1.75, Ni/CP-1.95 after stability test. Scan rate: 50 mV/s; electrolyte: 1 ethanol and 1 M KOH; IR compensated. The solid curves represent the forward scan and the dash curve indicate the backward scan.

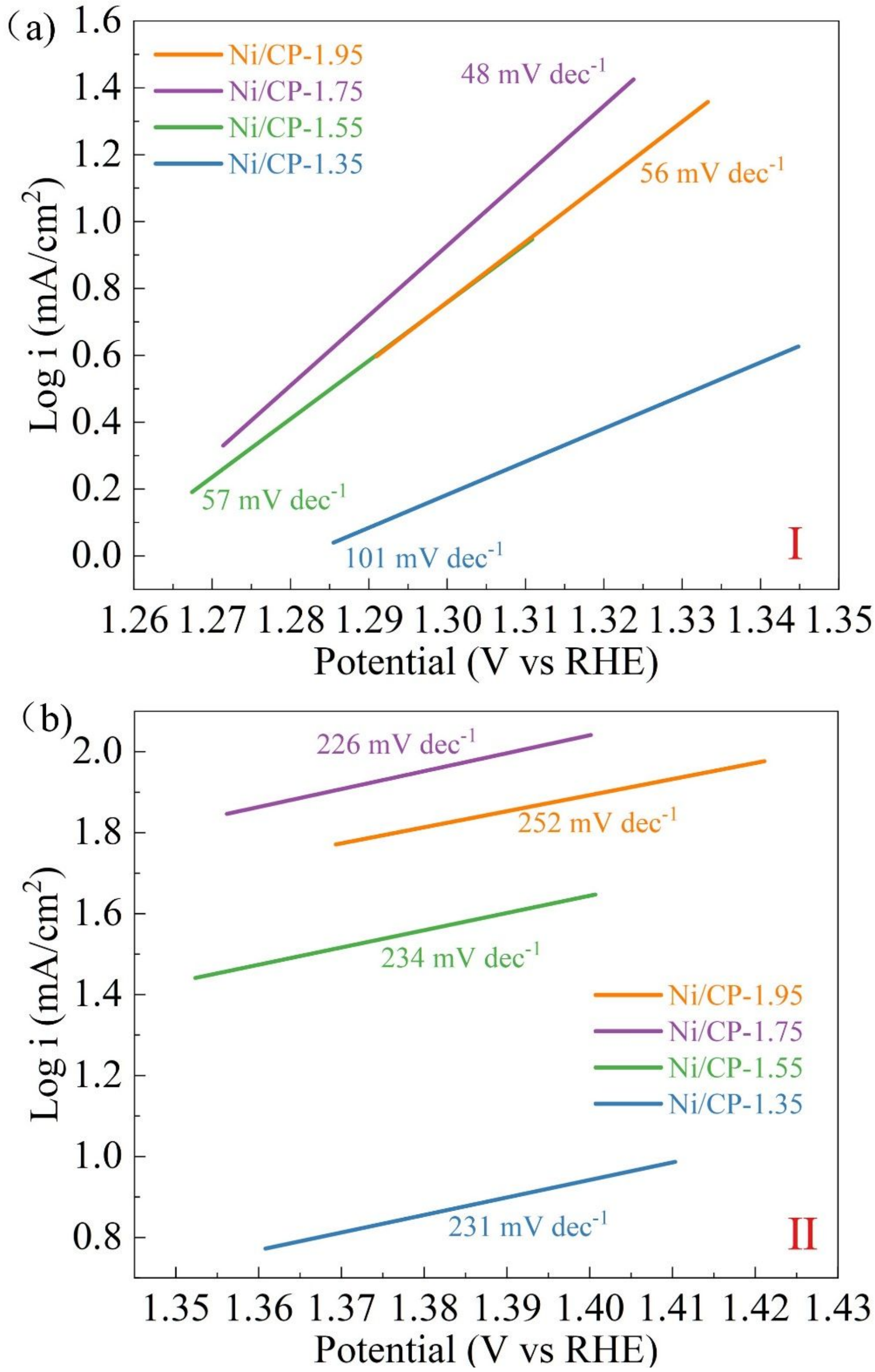

Figure S7. Tafel plots in range I (a) and II (b) over Ni/CP-1.35, Ni/CP-1.55, Ni/CP-1.75, and Ni/CP-1.95 electrode after stability test. Scan rate: 50 mV/s; IR compensated; electrolyte: 1 M ethanol and 1 M KOH solution.

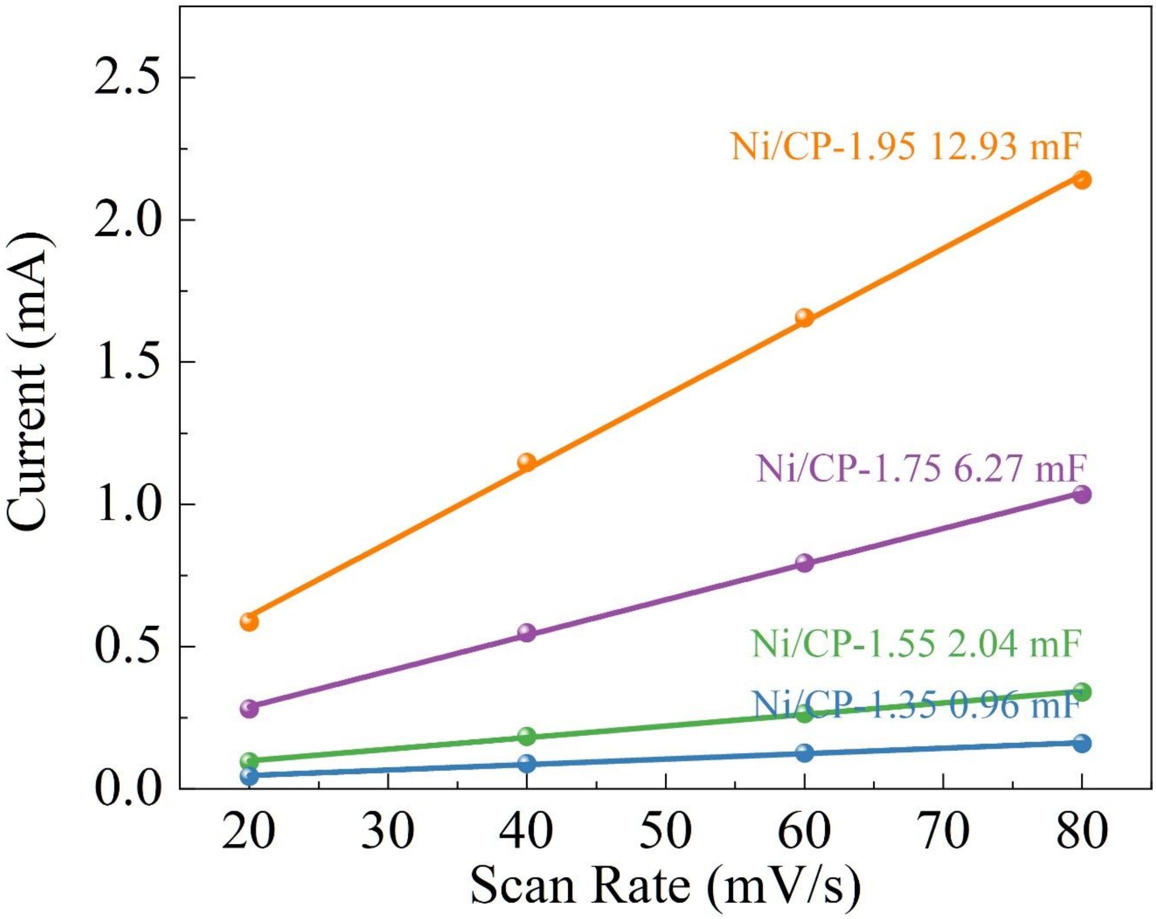

Figure S8. Scan rate dependence of the current over Ni/CP-1.35, Ni/CP-1.55, Ni/CP-1.75 and Ni/CP-1.95 after stability test.

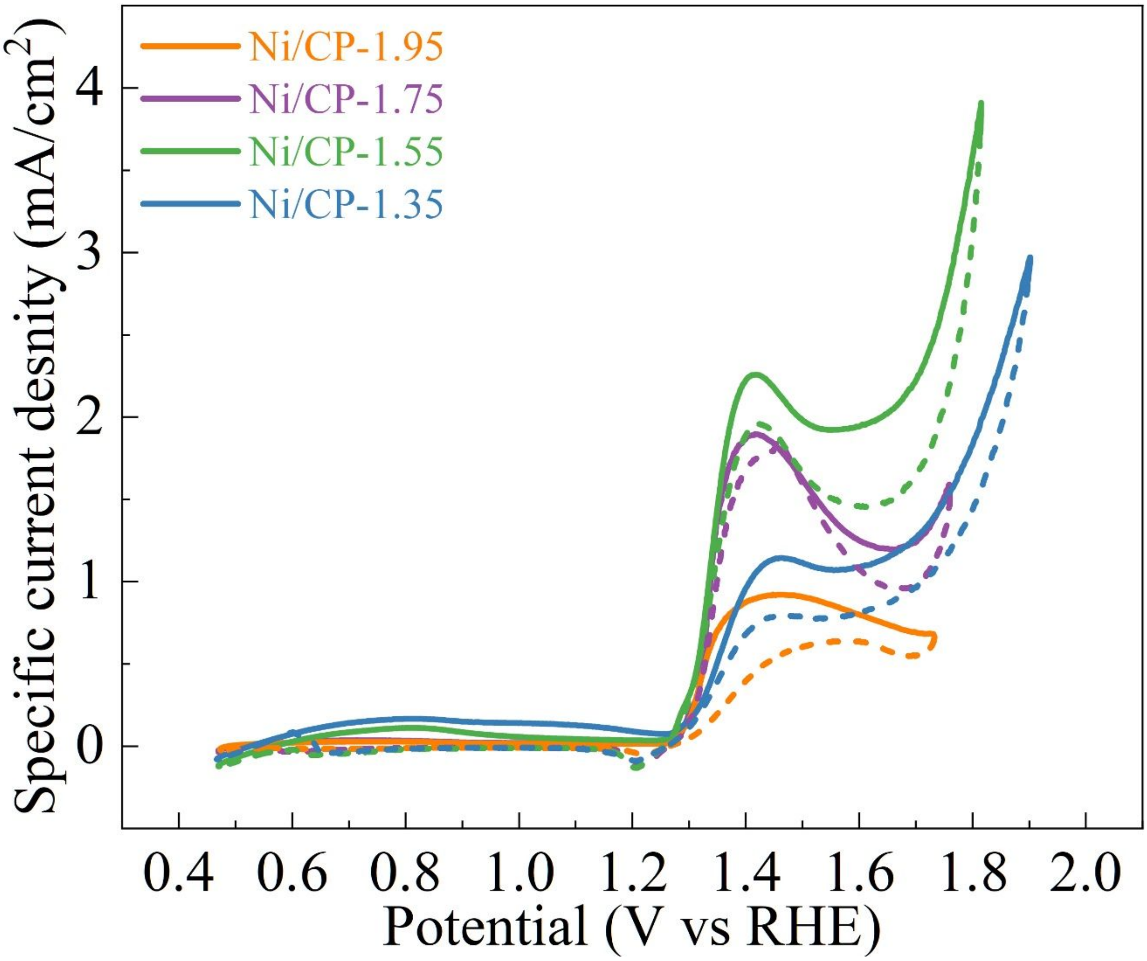

Figure S9. ECSA based CV curves of CP, Ni/CP-1.35, Ni/CP-1.55, Ni/CP-1.75, Ni/CP-1.95 after stability test. Scan rate: 50 mV/s; electrolyte: 1 ethanol and 1 M KOH; IR compensated. The solid curves represent the forward scan and the dash curve indicate the backward scan.

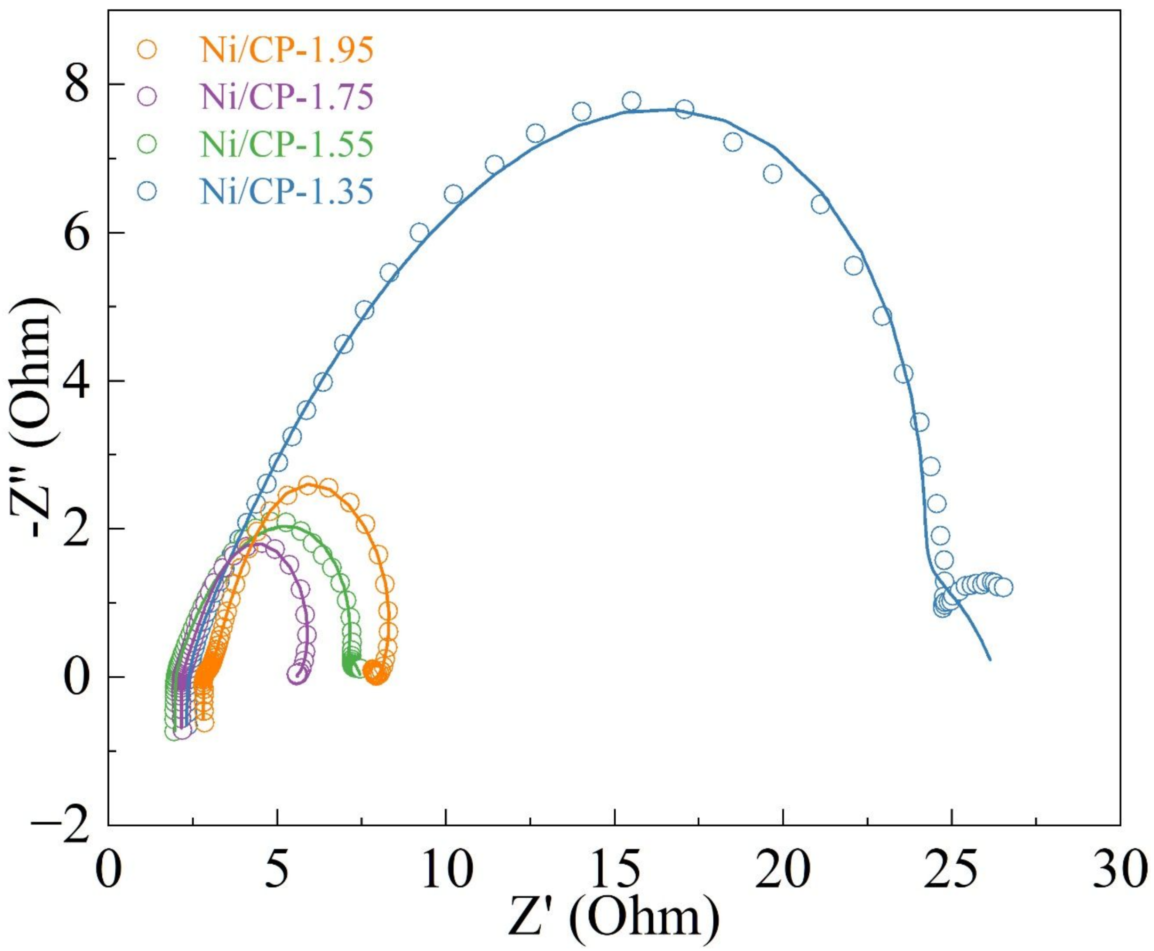

Figure S10. Nyquist plots with fit curves of Ni/CP electrodes deposited at various potentials after stability test

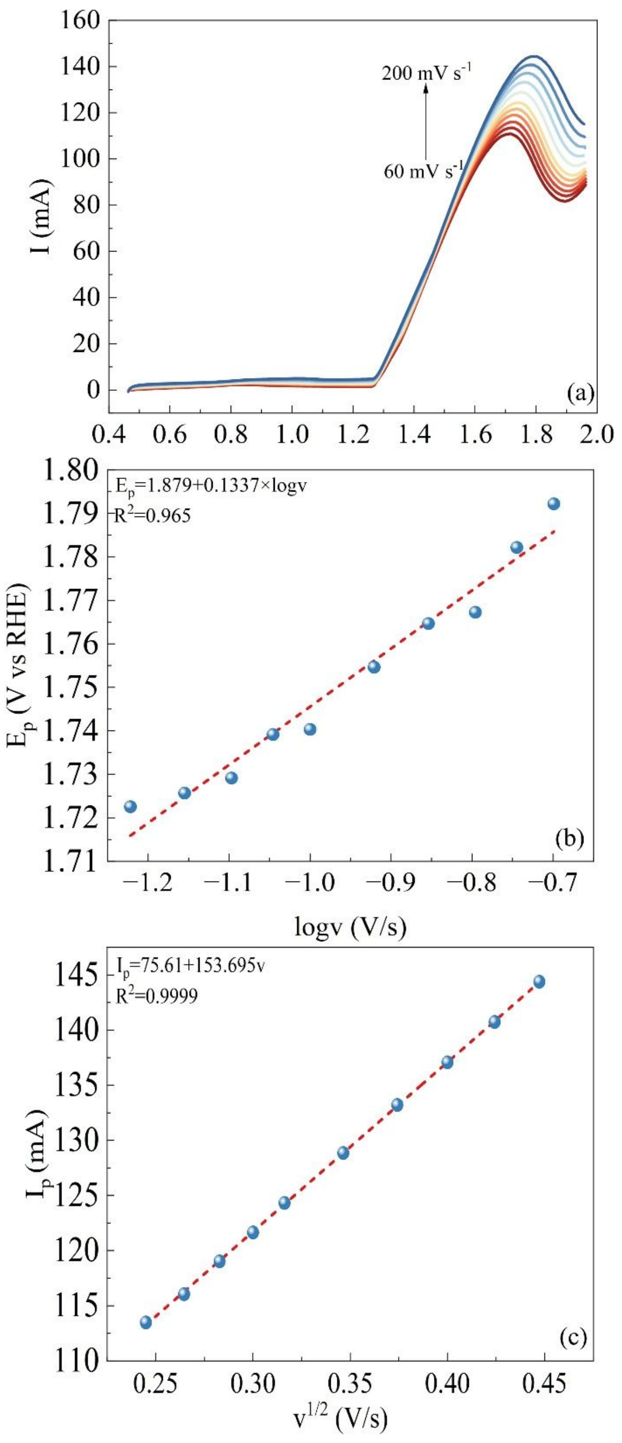

Figure S11. (a) Forward scans of CV curves obtained over Ni/CP-1.75 (after stability test) in 1 M KOH +1M ethanol with various scan rates of 60, 70, 80, 90, 100, 120, 140, 160, 180, and 200 mV/s; (b) Dependence of the peak potential (E_p) on the natural logarithm of the scan rate, log v, with linear fitting results; (c) Dependence of the peak current (I_p) on the square root of the scan rate ($v^{1/2}$) with linear fitting results.

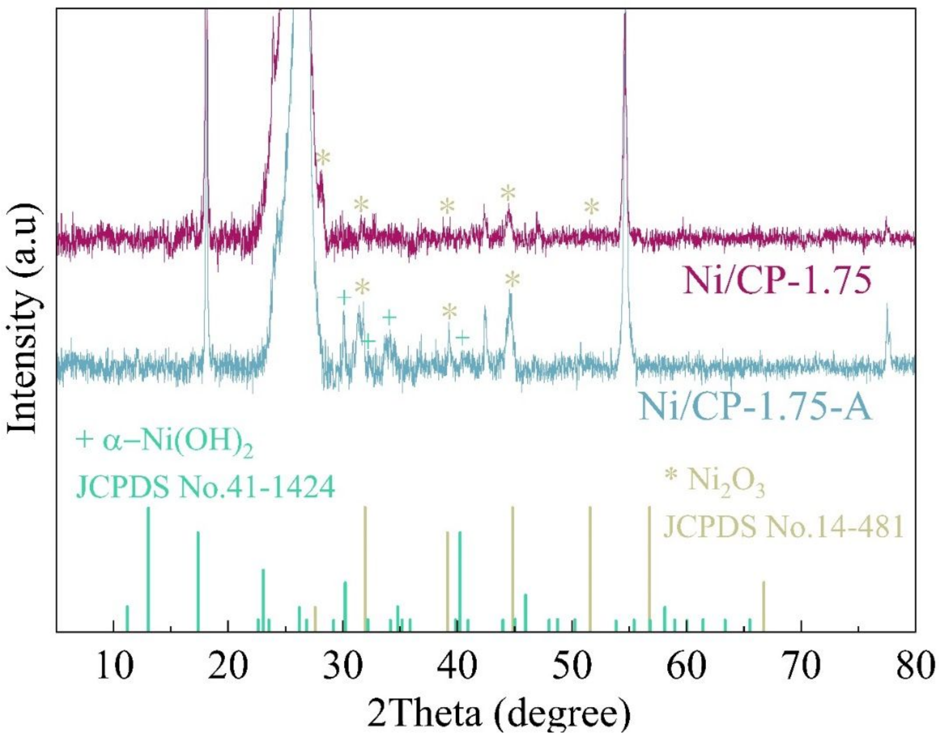

Figure S12. XRD patterns of Ni/CP-1.75 and after stability test.

Table S1. Comparison of peak current density for ethanol oxidation in alkaline medium with state of art Ni based catalysts.

Catalysts	j_p[a] mA cm^{-2}	P_p[b]	c_{EtOH}[c] M	SR[d] mV s^{-1}	Ref.
Ni-CNPs	47.0	0.75 V vs Ag/AgCl	1.0	50	[1]
$Ni(OH)_2$ aerogel	27.6	0.7 V vs Hg/HgO	0.1	50	[2]
Ni/gCH(H)	23.3	0.66 V vs Ag/AgCl	3.0	50	[3]
Ni/aHC	28.5	0.71 V vs Hg/HgO	1.0	50	[4]
NiAl-LDH-NSs	45.8	0.58 V vs Ag/AgCl	1.0	50	[5]
Ni-Co/graphene/GCE	16.4	0.71 V vs SCE	0.1	50	[6]
NiF/AuNPs	6.4	0.23 V vs Ag/AgCl	1.0	50	[7]
NiNC-3	47.5	0.51 V vs SCE	0.5	50	[8]
Ni_2O_3/CP	106.9	1.46 V vs RHE	1.0	50	This work

[a] peak current density; [b] peak potential; [c] ethanol concentration; [d] scan rate.

References

(1) Barakat, N. A. M.; Moustafa, H. M.; Nassar, M. M.; Abdelkareem, M. A.; Mahmoud, M. S.; Almajid, A. A.; Khalil, K. A. Distinct influence for carbon nano-morphology on the activity and optimum metal loading of Ni/C composite used for ethanol oxidation. *Electrochim. Acta* **2015**, *182*, 143-155.

(2) Zhou, X.-C.; Yang, X.-Y.; Fu, Z.-B.; Yang, Q.; Yang, X.; Tang, Y.-J.; Wang, C.-Y.; Yi, Y. Single-crystalline ultrathin nanofilms of Ni aerogel with Ni(OH)2 hybrid nanoparticles towards enhanced catalytic performance for ethanol electro-oxidation. *Appl. Surf. Sci.* **2019**, *492*, 756-764.

(3) Lewalska-Graczyk, A.; Pieta, P.; Garbarino, G.; Busca, G.; Holdynski, M.; Kalisz, G.; Sroka-Bartnicka, A.; Nowakowski, R.; Naushad, M.; Gawande, M. B. J. A. s. c.; et al. Graphitic carbon nitride–nickel catalyst: from material characterization to efficient ethanol electrooxidation. **2020**, *8* (18), 7244-7255.

(4) Cuña, A.; Plascencia, C. R.; da Silva, E. L.; Marcuzzo, J.; Khan, S.; Tancredi, N.; Baldan, M.; de Fraga Malfatti, C. J. A. C. B. E. Electrochemical and spectroelectrochemical analyses of hydrothermal carbon supported nickel electrocatalyst for ethanol electro-oxidation in alkaline medium. **2017**, *202*, 95-103.

(5) Xu, L.; Wang, Z.; Chen, X.; Qu, Z.; Li, F.; Yang, W. J. E. a. Ultrathin layered double hydroxide nanosheets with Ni (III) active species obtained by exfoliation for highly efficient ethanol electrooxidation. **2018**, *260*, 898-904.

(6) Wang, Z.; Du, Y.; Zhang, F.; Zheng, Z.; Zhang, Y.; Wang, C. J. J. o. s. s. e. High electrocatalytic activity of non-noble Ni-Co/graphene catalyst for direct ethanol fuel cells. **2013**, *17*, 99-107.

(7) Hatamie, A.; Rezvani, E.; Rasouli, A. S.; Simchi, A. J. E. Electrocatalytic Oxidation of Ethanol on Flexible Three-dimensional Interconnected Nickel/Gold Composite Foams in Alkaline Media. **2019**, *31* (3), 504-511.

(8) Shi, W.; Gao, H.; Yu, J.; Jia, M.; Dai, T.; Zhao, Y.; Xu, J.; Li, G. J. E. A. One-step synthesis of N-doped activated carbon with controllable Ni nanorods for ethanol oxidation. **2016**, *220*, 486-492.
